# Expanding the clinical and immunological phenotypes of PAX1-deficient SCID and CID patients

**DOI:** 10.1016/j.clim.2023.109757

**Published:** 2023-10

**Authors:** Nalan Yakici, Alexandra Y. Kreins, Mehmet Cihangir Catak, Royala Babayeva, Baran Erman, Heather Kenney, Hatice Eke Gungor, Pablo A. Cea, Tomoki Kawai, Marita Bosticardo, Ottavia Maria Delmonte, Stuart Adams, Yu-Tong Fan, Francesca Pala, Ayberk Turkyilmaz, Evey Howley, Austen Worth, Hakan Kot, Asena Pinar Sefer, Altan Kara, Alper Bulutoglu, Sevgi Bilgic-Eltan, Melek Yorgun Altunbas, Feyza Bayram Catak, Ibrahim Serhat Karakus, Emrah Karatay, Sidem Didar Tekeoglu, Metin Eser, Davut Albayrak, Senol Citli, Ayca Kiykim, Elif Karakoc-Aydiner, Ahmet Ozen, Sujal Ghosh, Holger Gohlke, Fazil Orhan, Luigi D. Notarangelo, E. Graham Davies, Safa Baris

**Affiliations:** a Department of Pediatrics, Division of Pediatric Allergy and Immunology, Faculty of Medicine, Karadeniz Technical University Trabzon, Turkey; b Great Ormond Street Institute of Child Health, Infection, Immunity and Inflammation Research & Teaching Department, University College London, London, United Kingdom; c Department of Immunology and Gene therapy, Great Ormond Street Hospital for Children NHS Foundation Trust, London, United Kingdom; d SIHMDS-Haematology, Great Ormond Street Hospital for Children NHS Foundation Trust, London, United Kingdom; e Division of Pediatric Allergy and Immunology, School of Medicine, Marmara University, Istanbul, Turkey; f Istanbul Jeffrey Modell Diagnostic and Research Center for Primary Immunodeficiencies, Istanbul, Turkey; g The Isil Berat Barlan Center for Translational Medicine, Istanbul, Turkey; h Institute of Child Health, Hacettepe University, Ankara, Turkey; i Can Sucak, Research Laboratory for Translational Immunology, Center for Genomics and Rare Diseases, Hacettepe University, Ankara, Turkey; j Immune Deficiency Genetics Section, Laboratory of Clinical Immunology and Microbiology (LCIM), National Institute of Allergy and Infectious Diseases (NIAID), NIH, Bethesda, MD, USA; k Division of Pediatric Allergy and Immunology, Erciyes City Hospital, Turkey; l Institute for Pharmaceutical and Medicinal Chemistry, Heinrich Heine University, Düsseldorf, Germany; m Shizuoka Children's Hospital, Shizuoka, Department of Allergy and Clinical Immunology, Japan; n Department of Medical Genetics, Faculty of Medicine, Karadeniz Technical University Trabzon, Turkey; o TUBITAK Marmara Research Center, Gene Engineering and Biotechnology Institute, Gebze, Turkey; p School of Medicine, Marmara University, Istanbul, Turkey; q Department of Radiology, Marmara University Pendik Training and Research Hospital, Istanbul, Turkey; r Department of Pediatric Immunology, Hacettepe University, Ankara, Turkey; s Department of Medical Genetics, Umraniye Education and Research Hospital, University of Health Sciences, Istanbul, Turkey; t Department of Pediatrics, Division of Pediatric Hematology, Medicalpark Hospital, Samsun, Turkey; u Department of Medical Genetics, Faculty of Medicine, Recep Tayyip Erdogan University, Rize, Turkey; v Department of Pediatrics, Division of Pediatric Allergy and Immunology, Faculty of Medicine, Istanbul University-Cerrahpasa, Istanbul, Turkey; w Department for Pediatric Oncology, Hematology and Clinical Immunology, Medical Faculty, Center of Child and Adolescent Health, Heinrich Heine University, Düsseldorf, Germany; x Institute of Bio- and Geosciences (IBG-4: Bioinformatics), Forschungszentrum Jülich GmbH, Jülich, Germany

**Keywords:** PAX1, Inborn errors of immunity, SCID, thymus, Otofaciocervical syndrome, Hypoparathyroidism

## Abstract

Paired box 1 (PAX1) deficiency has been reported in a small number of patients diagnosed with otofaciocervical syndrome type 2 (OFCS2). We described six new patients who demonstrated variable clinical penetrance. Reduced transcriptional activity of pathogenic variants confirmed partial or complete PAX1 deficiency. Thymic aplasia and hypoplasia were associated with impaired T cell immunity. Corrective treatment was required in 4/6 patients. Hematopoietic stem cell transplantation resulted in poor immune reconstitution with absent naïve T cells, contrasting with the superior recovery of T cell immunity after thymus transplantation. Normal *ex vivo* differentiation of PAX1-deficient CD34^+^ cells into mature T cells demonstrated the absence of a hematopoietic cell-intrinsic defect. New overlapping features with DiGeorge syndrome included primary hypoparathyroidism (*n* = 5) and congenital heart defects (*n* = 2), in line with PAX1 expression during early embryogenesis. Our results highlight new features of PAX1 deficiency, which are relevant to improving early diagnosis and identifying patients requiring corrective treatment.

## Highlights

• PAX1 deficiency shares new overlapping features with DiGeorge syndrome, including immunodeficiency and hypoparathyroidism.
• Partial or complete PAX1 deficiency results in clinical and immunological heterogeneity among patients.
• Clinical heterogenicity requires distinct therapeutic management like thymus transplantation.
• Expanding the disease spectrum provides early diagnosis and optimizes the treatment.

## 1 Introduction

Otofaciocervical syndrome (OFCS) is a rare disorder characterized by ear anomalies, including cup-shaped low-set ears, preauricular fistulas and hearing loss, facial dysmorphism, branchial defects, skeletal anomalies, and mild intellectual disability. To date, OFCS has been associated with two different genetic etiologies. Heterozygous mutations in *EYA1* gene have been identified in autosomal dominant cases, diagnosed with OFCS type 1 [1]. More recently, bi-allelic mutations in another gene, paired box 1 (*PAX1*), were identified in autosomal recessive cases from a large consanguineous family with OFCS, constituting a 2^nd^ type of OFCS (OFCS2) [2]. Since then, fourteen PAX1-deficient patients from seven unrelated kindreds have been reported [[2], [3], [4], [5], [6]]. PAX1 is a member of the paired box (PAX) family of transcription factors, playing a role in patterning during vertebrate embryogenesis [7]. In general, all nine *PAX* genes support embryonic cell proliferation, migration, survival, and cellular differentiation [8]. During early embryogenesis, PAX1 is expressed in the sclerotome as well as in 3^rd^ pharyngeal pouch (PP) derivatives, respectively giving rise to the skeletal system, thymus, tonsils, parathyroid and thyroid glands, and middle ear [7,9,10]. PAX1-deficient patients display all the hallmark OFCS features, while only mild clinical phenotypes, specifically ear anomalies, including pre-auricular pits, have been variably described in heterozygous relatives [3]. In contrast to OFCS1, OFCS2 has also been associated with significant immunodeficiency in reported patients [5]. The most severe cases displayed a T^−^B^+^NK^+^ severe combined immunodeficiency (SCID)-like phenotype secondary to thymic aplasia due to impaired differentiation of thymic epithelial cells [5]. PAX1 deficiency has since been classified as an inborn error of immunity characterized by combined immunodeficiency (CID) [11,12].

Inborn errors of thymic stromal cell development have been associated with defects in other transcription factors known to regulate early 3^rd^ PP patterning, including TBX1, TBX2, FOXI3, and CHD7 [13]. These defects have been identified in syndromic patients with overlapping clinical features, including thymic hypoplasia, hypoparathyroidism, and congenital heart disease, due to the common 3^rd^ PP-derived organogenesis of these affected systems [13]. These problems are a triad of hallmark features of DiGeorge syndrome (DGS). DGS is most commonly encountered in the context of 22q11.2 deletion syndrome. Regardless of the underlying genetic etiology, DGS has been associated with variable clinical penetrance across the different affected systems, including a broad spectrum of immunodeficiency. Patients with complete DGS (cDGS) have congenital athymia and require corrective treatment, ideally with thymus transplantation (TT) [14,15]. Other than thymic aplasia/hypoplasia, no overlapping features have been reported between OFCS2 and DGS patients. So far, only seven OFCS2 kindreds have been reported and incompletely described in the literature, with scant information available about the multi-system and immunological consequences of the disease [[2], [3], [4], [5], [6]]. Herein, we present five new PAX1-deficient patients and provide additional clinical information on a sixth patient whose mutation was recently reported [6]. These cases originate from six unrelated kindreds, all presented with CID or SCID. Our study provides a more detailed clinical and immunological characterization expanding the spectrum of disease associated with PAX1 deficiency, with important clinical implications to facilitate early diagnosis and treatment.

## 2 Material and methods

### 2.1 Patients

This multi-center study includes six PAX1-deficient patients from unrelated kindreds. Genetic diagnosis was made by whole-exome sequencing (WES) and confirmed by Sanger sequencing [16,17]. The local ethics committee from Karadeniz University approved the study protocol for patients treated in Turkey, and the London Bloomsbury Research Ethics Committee approved the studies for patients treated at Great Ormond Street Hospital in London, UK. NIH studies were conducted according to protocol 18-I-0041 (registered as NCT03610802 at www.clinicaltrial.gov). Written informed consent was obtained from all families. Clinical and laboratory data were retrieved retrospectively from clinical records. Completed clinical questionnaires included information on demographics, clinical presentation at diagnosis and during follow-up, and treatment outcomes. Laboratory information contained full blood counts, IgG, IgA, and IgM levels, lymphocyte subsets with CD3^+^, CD4^+^, CD8^+^, naïve CD4^+^CD45RA^+^ and CD8^+^CD45RA^+^ T cells, CD4^+^CD31^+^CD45RA^+^ recent thymic emigrants (RTE), CD19^+^ B cells, CD16^+^CD56^+^ NK cells, mitogen-induced proliferative response, and T cell receptor (TCR) Vβ repertoire. Peripheral blood lymphocyte subset analyses, upregulation and proliferation assays were performed by flow cytometry as described previously [16,18,19]. Details are provided in the **Repository file**.

### 2.2 Structural analysis of PAX1 variants

The boundaries between domains of *PAX1* and the secondary structure were predicted with TopDomain [20] and TopProperty [21], respectively, on the sequence with UniProt ID P15863. Structural modelling of the Paired Box Domain (PBD) was carried out using TopModel [22]. Multiple Sequence alignments were constructed using HMMER [23]. Co-evolutionary analyses were performed using GREMLIN [24].

### 2.3 Generation of *Pax1* constructs

*Pax1* mouse mutant variants (*Pax1*^*L64P*^*, Pax1*^*S281X*^*, Pax1*^*A327fs⁎15*^*, Pax1*^*P153L*^*, Pax1*^*S159A*^*)* were generated by site-directed mutagenesis in a pCMV-HAN vector (Addgene, Cambridge, MA) containing the wild type mouse *Pax1*-coding sequence (NM_008780.2), as described [2,5,25]. PCR products were digested with DPN1 (NEB, Ipswich, MA), ligated with Quick Ligation Kit (NEB) and cloned into 5-alpha F'*I*^*q*^ Competent *E. coli* High-Efficiency cells (Promega). The cDNA sequences of the constructs were confirmed by Sanger sequencing.

### 2.4 Luciferase reporter assay for analysis of Pax1 transcriptional activity

293 T cells were cultured and co-transfected with 30 ng of either wild type (WT) or mutant *Pax1* expression plasmids, 15 ng firefly reporter plasmid *Nkx3–2*-pGL4.10luc2, and 3 ng of pRL-TK vector (Promega) for normalization as previously described [5]. After 48 h, cell lysates were collected, and *firefly* and *renilla* luciferase activities were measured using a Dual-Luciferase Reporter Assay kit (Promega) and Filter-based multi-mode microplate reader, FLUOstar Omega (BMG Labtech). Details are provided in the **Repository file**. The luciferase activity with the empty vector (which had no *Pax1* cDNA) was set at 0% and the activity with the WT construct at 100%.

### 2.5 T cell receptor beta (TRB) repertoire analyses

CD4^+^ and CD8^+^ T cells were isolated from peripheral blood mononuclear cells (PBMCs) of patients and healthy controls by a negative magnetic separation system (BioLegend). Total RNA was extracted using the NucleoSpin RNA purification kit (Macherey-Nagel). 50 ng bulk RNA samples were used as starting material for TCR profiling. Repertoire libraries were prepared by SMARTer® Human TCR α/β Profiling Kit (Takara). Details are provided in the **Repository file**. For P5, T cell clonality was assessed using TCRVβ chain spectratyping on isolated CD3^+^ T cells as previously described [26].

### 2.6 *In vitro* T cell differentiation study

*In vitro* T cell differentiation was studied by co-culturing CD34^+^ cells isolated from PBMCs with a DLL4-expressing stromal cell line (MS5-hDLL4) in an artificial thymic organoid (ATO) system as previously described with minor modifications [27]. Details are provided in the **Repository file**.

### 2.7 Statistical analysis

The data is expressed with percentage, median with a minimum-maximum range, and mean with standard error of the mean, as indicated. Analysis of luciferase reporter activity was carried out by one-way ANOVA and adjusted by Bonferroni-Dunn multiple comparisons test. Differences in values were considered significant at a *p*-value <0.05

## 3 Results

### 3.1 Diagnosis of PAX1 deficiency

**Fig. 1:**
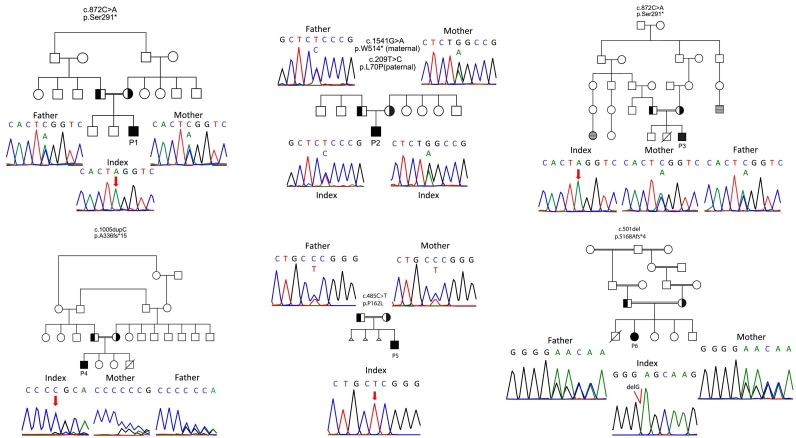
Family pedigrees of patients. Pedigrees and results of Sanger sequencing in patients with PAX1 variants. Double lines indicate consanguinity; filled black circles or squares depict the patients; half-filled black circles or squares depict the carrier state; diagonal lines indicate deceased individuals. Gray dashed circles or squares indicate individuals with ear abnormalities and hearing loss. Triangles indicate miscarriages. Males and females are distinguished by squares and circles, respectively.

Six syndromic patients (P1–6) from unrelated kindreds (Fig. 1) were diagnosed with genetically undefined T^-/low^B^+^NK^+^ SCID or CID. All six patients displayed facial dysmorphism, ear and skeletal abnormalities compatible with OFCS. Detailed case reports are available in the **Repository file**, and their clinical features are summarized in Table 1. Four patients (P1–4) presented clinically within the first year of life with infections (P1, P3), Omenn-like symptoms (P1), hypocalcemia (P1–4), or autoimmune cytopenias (P4). The remaining two patients were identified at birth. P5 was identified following absent T cell receptor excision circles (TREC) in newborn screening for SCID [28], and P6 was assessed at birth in the context of a positive family history for suspected leaky SCID in a previous infant who died at 3 months old due to RSV pneumonia. P6 also presented neonatal hypocalcemia. All families had parental consanguinity except for P2. Family history revealed that P3 also had an older sibling with syndromic features who died at 18 months of pneumonia and respiratory failure. One patient (P3) died at the age of 21 months due to EBV-positive lymphoma. The other five patients are alive with a median follow-up of 2.3 years (min-max: 1.2–11.9) at the time of completion of data collection. The deceased siblings of P3 and P6 were not included in the study, as their genetic status remains unconfirmed.

**Table 1:** The demographics, clinical features, and outcome of the PAX1-deficient patients.

Patient	Total (n,%)	P1	P2	P3	P4	P5	P6
*Last documented age (mo)/Sex*	83% M, 17% F	21/M	23/M	21/M	144/M	36/M	144/F
*Consanguinity*	83%	+	+	−	+	+	+
*Age at onset (mo)*	Median: 1	1	1	6	1	1	1
*Growth reterdation*	67%	No	No	Yes	Yes	Yes	Yes
*Developmental delay*	50%	Yes	No	No	Yes	Yes	No
*Intellectual disability*	67%	Yes-Mild	No	Yes-Mild	Yes-Moderate	Yes-mild	No

*Dysmorphic features*							
Facial asymmetry	67%	Yes	No	No	Yes	Yes	Yes
Microtia	83%	Yes	No	Yes	Yes	Yes	Yes
Low-set ears	100%	Yes	Yes	Yes	Yes	Yes	Yes
Preauricular pits	50%	No	No	No	Yes	Yes	Yes
Nasal root flattening	100%	Yes	Yes	Yes	Yes	Yes	Yes
Broad forehead	34%	Yes	Yes	No	No	No	No
Hypertelorism	83%	Yes	Yes	Yes	No	Yes	Yes
Epicanthus	67%	Yes	Yes	Yes	Yes	No	No
Down slanting palpebral fissures	33%	Yes	No	Yes	No	No	No
Anteverted nostrils	33%	Yes	No	Yes	No	No	No
Hypoplastic alae nasi	33%	No	No	Yes	No	No	Yes
Long philtrum	17%	No	Yes	No	No	No	No
Flat philtrum	17%	No	No	No	Yes	No	No
Tented philtrum	17%	Yes	No	No	No	No	No
Thin upper lip	67%	Yes	Yes	Yes	No	No	Yes
Micrognathia	50%	Yes	Yes	Yes	No	No	No
Retrognathia	33%	Yes	No	Yes	No	Yes	No
Short neck	34%	No	No	Yes	Yes	No	No
Undescended testices	40%	Yes	No	No	Yes	No	Not-applicable

*Skeletal abnormalities*							
Hooked distal clavicles	67%	Yes	No	Yes	Yes	No	Yes
Split vertebral bodies	17%	Yes	No	No	No	No	No
Hypoplastic vertebrae	67%	No	Yes	No	Yes	Yes	Yes
Butterffly vertebrae	67%	Yes	No	Yes	Yes	No	Yes
Kyphosis	67%	Yes	No	No	Yes	Yes	Yes
Scoliosis	67%	Yes	Yes	No	No	Yes	Yes
Winged scapula	17%	No	No	No	Yes	No	No
*Hearing loss*	83%	Yes	No	Yes	Yes	Yes	Yes
*Immunodeficiency*	100%	Yes	Yes	Yes	Yes	Yes	Yes
*Thymic aplasia/hypoplasia*	83%	Yes-Aplastic	Yes-Rudimental	Yes-Aplastic	Yes-Aplastic	Yes-Aplastic	Yes-Rudimental
Hypoparathyroidism	83%	Yes-Persistent	Yes-Transient	Yes-Persistent	Yes-Persistent	No	Yes-Persistent
Hypothyroidism	17%	No	Yes	No	No	No	No
*Cardiac malformation*	33%	No	Yes	Yes	No	No	No
*Infections*	50%	Yes	No	Yes	No	No	Yes
*Eczema*	17%	Yes	No	No	No	No	No
*Autoimmunity*	50%	No	Yes	Yes	Yes	No	No
*Thymus transplantation*	17%	No	No	No	No	Yes	No
*HSCT*	33%	Yes	No	No	No	No	Yes
*Outcome (alive)*	83%	Alive	Alive	Dead-EBV (+) diffuse large cell lymphoma	Alive	Alive	Alive

**Fig. 2:**
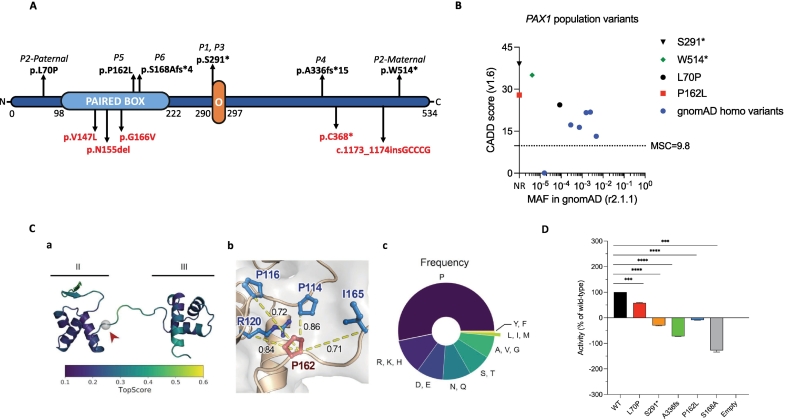
Variants in PAX1 deficiency are distributed throughout the gene. **(A) (A)** Schematic diagram of PAX1 protein domains with variants. Asterisk (*) indicates a premature stop codon. Previously reported mutations are shown by red color. **(B)** The PAX1 variants with allele frequencies and CADD scores (NR: Not-reported). **(C)** Analysis of the P162L variant located in the paired box domain of PAX1. **a)** Structural model of the paired box domain, highlighting the two helical bundles (II and III). The structure was colored according to the model quality score TopScore (scores close to 0 indicate a high-quality model and scores close to 1 a low-quality model). The position of the variant is highlighted with a gray sphere and a red arrowhead. **b)** Co-evolutionary network of P162 (shown in red). Co-evolving residues are shown in blue. The pair-wise co-evolutionary coupling scores are shown over yellow dashed lines (score of 1.0 means perfectly coupled, and 0 means not coupled). **c)** Residue distribution found at position 162 on a multiple sequence alignment of PAX1 homologs. The residues are grouped according to their physicochemical properties (P: proline (WT residue). R; K; H: positively charged. D, E: negatively charged. N, Q: long polar. S, T: short polar. A, V, G: small aliphatic. L, I, M: large aliphatic. Y, F: aromatic). **(D)** Luciferase assay showing reduced transcriptional activity of mutant PAX1 proteins, corresponding to the *PAX1* variants detected in patients. The promoter region of Nkx3–2 was used to drive luciferase expression. Results of 3 independent experiments are presented (means ± SEM). *P* value was calculated with one-way ANOVA and adjusted by Bonferroni-Dunn multiple comparisons test. ***P < 0.001; ****P < 0.0001. (For interpretation of the references to color in this figure legend, the reader is referred to the web version of this article.)

P1–6 were investigated by WES and were found to carry homozygous or compound heterozygous mutations in *PAX1*. Six different mutations were identified, including a homozygous stop-gain mutation identified in P1 and P3 (c.872C > A, p.S291*), a maternally inherited stop-gain mutation (c.1541G > A, p.W514*) and a paternally inherited missense mutation (c.209 T > C, p.L70P) in P2, homozygous frameshift mutations in P4 (c.1005dupC, p.A336fs*15) and P6 (c.501del, p.S168Afs*4), and a homozygous missense mutation in P5 (c.485C > T, p.P162L) (Fig. 1). p.S291* was identified in two unrelated patients (P1, P3) with origins in the same geographical region, suggesting it is a founder mutation. The mutations and affected domains of the PAX1 protein are depicted in Fig. 2A and Fig.E1A. These variants have not been reported in any public databases except for p.W514* and p.L70P, which are both extremely rare (Fig. 2B, Table E1). Apart from W514*, all the variants affect highly conserved amino acids, as demonstrated by multiple sequence alignment (Fig.E1B). Besides stop-gain and frameshift mutations, which are known to be destructive, high Combined Annotation Dependent Depletion (CADD) scores further suggested deleterious variants (Fig. 2B, Table E1). Altogether, a considerable pathogenic potential on protein structure for all six variants seemed likely.

### 3.2 Molecular modelling and analysis of the transcriptional activity of the PAX1 mutants

PAX1 is predominantly an intrinsically disordered protein with a single structured, helix-rich domain known as the PBD. We predicted the location of PAX1 domains along the sequence (Fig.E1A, upper plot) and the secondary structure elements that make up the protein and mapped the position of the six patient variants (Fig.E1A, lower plot). p.P162L and p.S168Afs*4 affect the PBD. p.L70P affects the protein's N-terminal intrinsically disordered region (IDR), whereas p.S291*, p.A336fs*15, and p.W514* affect the C-terminal IDR. The premature stop codon p.S291* results in a loss of ∼45% of the total protein length, likely abolishing the protein function, as this region is essential [29]. p.W514*, another premature stop codon, can also hamper proper protein function. The frameshift variants, p.S168Afs*4 and p.A336fs*15, are mutations that would disrupt over 68% and 37% of the regular reading frame, respectively. Frameshift mutations are known to be pathogenic in PAX2, another PBD-containing protein [30].

p.P162L is the only variant due to a missense mutation in the PBD. To study the putative effect of this substitution, we constructed a structural model of the PBD region of PAX1, which is formed by two helical bundles joined by an unstructured region, where the affected proline residue lies at the end of the first bundle (Fig. 2C, a). Next, we performed a co-evolutionary analysis, which can effectively identify disease-associated variants [31]. P162 strongly co-evolved with four adjacent residues, P114, P116, R120, and I165 (Fig. 2C, b). Disrupting a co-evolving residue network likely distorts the protein function [32]. To further support that P162L harms protein structure and/or function, we quantified the frequency of amino acid residues in the same position in other paired box homologous sequences. Mutations introducing residues frequently observed in this position should be less likely pathogenic, as they are tolerated by natural selection; by contrast, infrequently occurring residues are less likely to yield a functional protein. Proline is the most commonly observed residue in this position, present in over 53% of the cases, followed by polar amino acids (Fig. 2C, c). <1.2% of the analyzed sequences have large aliphatic amino acids in the position. Hence, L at this position is likely detrimental to protein function. It was not possible to perform similar analyses to study the putative effect of p.L70P due to a lack of depth of the multiple sequence alignment in this region.

To test the functional activity of the PAX1 variants, we relied upon a luciferase reporter assay in which the transcriptional activity of mouse Pax1 construct is measured on a target murine *Nkx3–2*-luciferase reporter plasmid. Upon cotransfection of the *Nkx3–2*-luciferase reporter plasmid and of either WT or mutant mouse *Pax1* expression plasmids (containing the orthologue variants observed in patients) into 293 T cells, analysis of luciferase activity showed that the cells overexpressing the p.P162L, p.S168A, p.S291*, and p.A336fs PAX1 mutant proteins had a similar transcriptional activity to that of cells transfected with the empty vector (set to 0%), indicating loss-of-function when compared with WT PAX1 (set to 100%) (Fig. 2D). Overexpression of certain variants, in particular, p.A336fs and p.S168A seem to further downregulate the luciferase activity to give a negative value in this assay, possibly through a competitive inhibitory effect on other transcription factors that regulate *Nkx3–2* promoter activity in PAX1-deficient 293 T cells. The p.L70P variant had reduced transcriptional activity that was approximately half that of wild-type PAX1. The transcriptional activity of W514* could not be tested because mouse Pax1 protein does not share the C-terminal region with human PAX1.

### 3.3 Clinical phenotypes of the patients

**Fig. 3:**
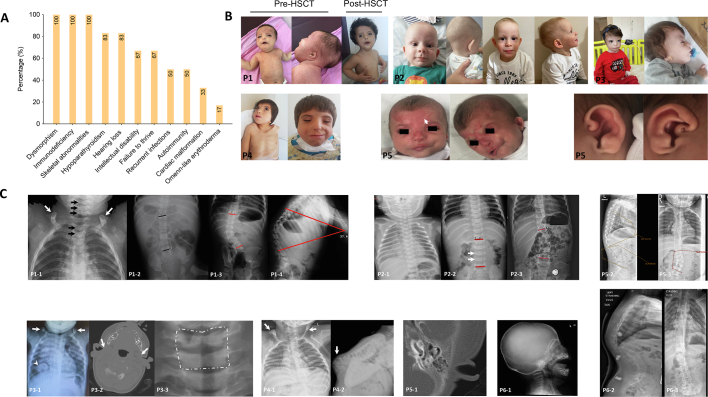
Clinical phenotypes of PAX1-deficient patients showing specific features. (A) The main manifestations of PAX1 deficiency. **(B)** Representative pictures of patients' phenotypes: **(P1)** Facial dysmorphisms (bilateral microtia, nasal root flattening, hypertelorism, epicanthus, and micrognathia), alopecia, and Omenn-like generalized rash (resolved with HSCT). **(P2)** Dysmorphisms characterized by low-set ears, short nose with low nasal bridge, hypertelorism, epicanthus, micrognathia, long philtrum, and thin upper lip. **(P3)** Micro- and retrognathia, maxillary hypoplasia, telecanthus, bilateral epicanthus, small and anteverted nostrils, microtia, low set ears, down slanting palpebral fissures, and thin upper lip. **(P4)** Facial dysmorphism including hypertelorism, flattened nasal root, epicanthus, flat philtrum, long eyelashes, right facial paralysis, bilateral preauricular cupping, microtia, short neck, and microdontia. **(P5)** Low-set ears with pre-auricular pits, overfolded ears, and a hypoplastic mandible with retrognathia. Flat nasal bridge with nevus flammeus (arrow) / asymmetric face. **(C)** Radiological findings of the patients. **P1–1**: Chest X-ray with no thymus shadow, butterfly (C4-T1) and split (T2-T4) vertebral bodies (black arrows), and hooked distal clavicles (white arrows). **P1–2** and **P1–3**: Increased lumber dextroscoliosis centralized at the level of L1 over time (Cobb angles: 5.2^°^ (black) *vs.* 33.3^°^ (red)). **P1–4**: Thoracolumbar kyphosis. **P2–1**: A rudimentary thymus shadow on chest X-ray. **P2–2**: Hypoplastic vertebrae (L4-L5) on X-ray spin (white arrows). **P2–2** and **P2–3**: Increased lumber levoscoliosis centralized at the level of L2 over time (Cobb angles (red lines): 1.8^°^*vs.* 18.8^°^). **P3–1**: Chest X-ray shows hooked distal clavicles (arrows) and multiple nodular opacities with pneumonic infiltration (arrow head). **P3–2**: Temporal bone computed tomography demonstrates bilateral external auditory canal atresia (white arrows). **P3–3**: C6 and C7 butterfly vertebrae on X-ray graph (white square). **P4–1**: Hooked distal clavicles (arrows). **P4–2**: Gibbus on chest X-ray graph (white arrows). **P5–1**: Aplastic or fully obstructed external auditory meatus, with no eardrum visible. Hypoplastic ossicles and increased sclerosis in the cochlear and semicircular canal region. **P5–2** and **P5–3**: Hyperkyphosis and lumbar levoscoliosis**. P6–1**: Prominent mid-face with beak-like appearance. **P6–2** and **P6–3**: Scoliosis on X-ray of the whole spine. **HSCT**: Hematopietic stem cell transplantation. (For interpretation of the references to color in this figure legend, the reader is referred to the web version of this article.)

Clinical manifestations are summarized in Table 1 and Fig. 3A. All patients presented with syndromic features corresponding to OFCS2, including facial dysmorphism (*n* = 6/6), skeletal abnormalities (n = 6/6), hearing loss (*n* = 5/6), intellectual disability (*n* = 4/6), and growth retardation (n = 4/6).

In particular, dysmorphic features were apparent at birth in all patients (Table 1 and Fig. 3B: P1-P5; dysmorphic features in P6 have been illustrated elsewhere [6]). Dysmorphic features were discrete in P2 but otherwise remained prominent over time in all the other patients. Low-set ears and nasal root flattening were observed in all patients. Microtia and hypertelorism were seen in 5/6 patients. Other common features were facial asymmetry (*n* = 4/6), epicanthus (n = 4/6), thin upper lip (n = 4/6), preauricular pits (*n* = 3/6), micrognathia (n = 3/6), and bilateral cryptorchidism (*n* = 2/5). Rarer features are detailed in Table 1. Hearing loss was detected in all patients except P2 (n = 5/6) and developmental delay in 3/6.

Concomitant to facial dysmorphism, skeletal surveys also demonstrated common skeletal abnormalities (Table 1 and Fig. 3C). Most patients showed hooked distal clavicles (n = 4/6), hypoplastic vertebrae formation (n = 4/6) and butterfly-shaped vertebrae (n = 4/6). Severe kyphosis was observed in 4/6 patients. Scoliosis was also seen in 4/6 patients, including overtime in P2, who had a normal initial skeletal survey. Skeletal abnormalities progressed in all patients, indicating the need for careful monitoring.

In summary, we find that all patients displayed syndromic features corresponding to OFCS but described variable clinical penetrance, as illustrated in particular by the overall mild syndromic features in P2.

### 3.4 Immunodeficiency in PAX1 deficiency

All patients presented with signs suggestive of (S)CID, including recurrent infections (n = 3/6), failure to thrive (n = 4/6), and Omenn-like diffuse erythroderma (*n* = 1/6). Autoimmune cytopenias were seen in 3/6 patients (Fig. 3A).

Three patients in our cohort were diagnosed with genetically undefined SCID; the other three with CID. All patients were started on antimicrobial prophylaxis and immunoglobulin replacement treatment (IgRT). Their immunological assessment is detailed below. Clinically, one SCID patient, P1, presented atypical features with an Omenn-like syndrome developing when 15 days old with rapidly worsening erythroderma complicated with seborrheic dermatitis and alopecia (Fig. 3B). He also suffered from chronic enteritis. Furthermore, three patients experienced recurrent infections (Table 1). Specifically, P1 and P6 had recurrent neonatal respiratory infections and pneumonia. Both patients were treated with hematopoietic stem cell transplantation (HSCT). P3 developed EBV viremia associated with pneumonia and axillary lymphadenopathy at 6 months old. Although copy numbers remained stable at approximately 280 copies/mL without specific treatment, at 19 months, he developed widespread pulmonary nodules, some of which showed cavitation (Fig. 3C). Histological examination of a resected lesion confirmed EBV-positive diffuse large cell lymphoma. He died at 21 months before treatment could be initiated. P5 did not suffer any significant infections before undergoing treatment with TT. Both CID patients, P2 and P4, did not suffer any significant infections either. P3 and P4 developed autoimmune hemolytic anemia, which resolved after treatment with high-dose intravenous immunoglobulins. P2 presented transient autoimmune neutropenia, which resolved spontaneously.

In summary, we report heterogeneity in terms of immunodeficiency, ranging from CID to SCID-like phenotypes, the latter requiring corrective treatment. We also report atypical features, including EBV-related lymphoproliferative disease in one patient and autoimmune manifestations in three patients.

### 3.5 Overlapping clinical features with DGS

The hallmark features of DGS are thymic hypoplasia/aplasia, primary hypoparathyroidism, and conotruncal heart defects. Using different imaging modalities (X-ray, ultrasound), no thymus tissue was seen in the mediastinum for P1, P3, P4, and P5, and only rudimental tissue was detected in P2 and P6. Residual but ectopic thymus tissue cannot fully be excluded by imaging. Therefore, it is best to rely on the assessment of thymic output (see below) to qualify thymic aplasia/hypoplasia.

Primary hypoparathyroidism was diagnosed in 5/6 patients (Table 1). Three patients presented with neonatal hypocalcemia; in the other two, hypoparathyroidism was diagnosed at twelve months. P2 was diagnosed after developing neonatal seizures, and P6 after neonatal tetany. In P1, P3, and P4, the diagnosis of hypoparathyroidism was incidental due to the documentation of low calcium levels in otherwise asymptomatic patients. All five patients were found to have low serum calcium levels with inappropriately low parathormone levels (PTH) (more details are presented in patients' summaries, **Repository file**). Vitamin D levels were normal in all patients. In P1, P3, and P4, hypoparathyroidism was persistent during follow-up, requiring continued calcium supplementation. In P2 and P6, hypocalcemia resolved spontaneously over time, and calcium supplementation was discontinued. Several years later, P6 again displayed tetany with low calcium and PTH levels. She has since been re-started on calcium supplementation. Apart from hypoparathyroidism, P2 was diagnosed with primary hypothyroidism and commenced on L-thyroxine treatment.

2/6 patients, P2 and P3, had atrial septal defects, which were not hemodynamically significant and did not require correction.

### 3.6 Detailed immunological evaluations

**Fig. 4:**
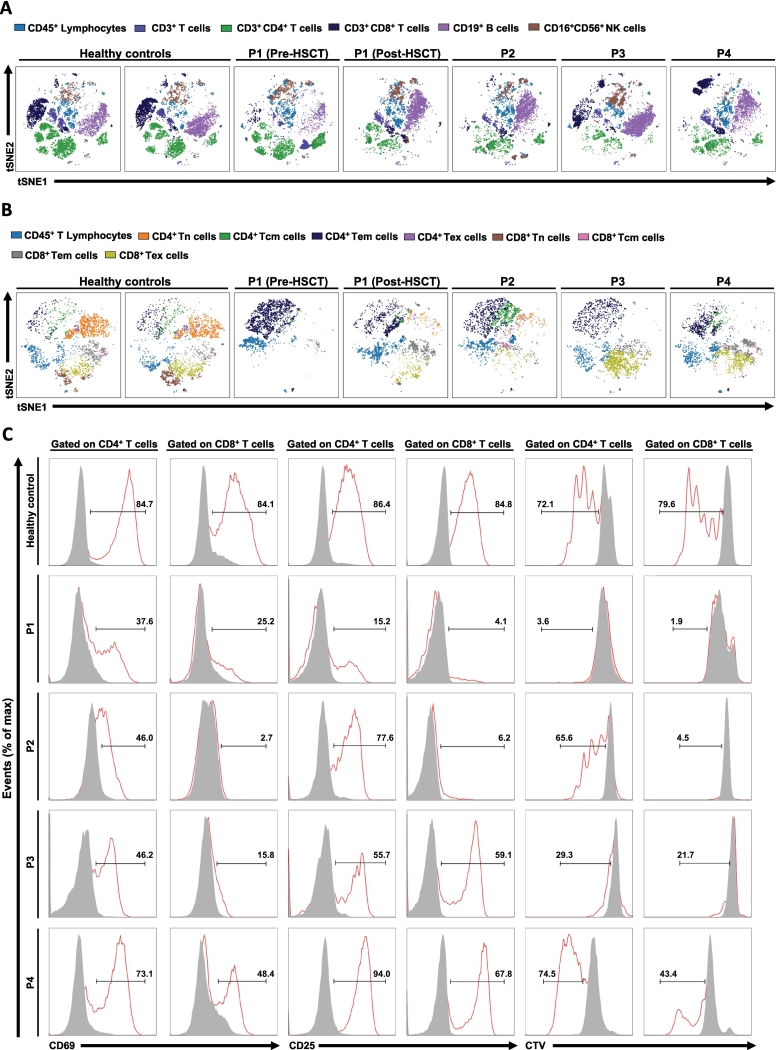
Diminished T cells and defective proliferation in PAX1 deficiency. (A) A dimensional reduction analysis showing the comparison of T, B, and NK cells between patients (P) and healthy controls (HC). **(B)** A dimensional reduction analysis showing the comparison of T cell subpopulations between patients (P) and healthy controls (HC). (**C**) Flow cytometric analysis of percentages of CD69, CD25, and proliferation in CD4^+^ and CD8^+^ T cells of patients and healthy controls in unstimulated and stimulated (anti-CD3 and anti-CD28) conditions. CD4^+^ naive T cells (Tn, CD4^+^CD45RA^+^CCR7^+^), central memory CD4^+^ T cells (Tcm, CD4^+^CD45RA^−^CCR7^+^), effector memory CD4^+^ T cells (Tem, CD4^+^CD45RA^−^CCR7^−^), terminally differentiated effector memory CD4^+^ T cells (Tex, CD4^+^CD45RA^+^CCR7^−^), CD8^+^ naive T cells (Tn, CD8^+^CD45RA^+^CCR7^+^), central memory CD8^+^ T cells (Tcm, CD8^+^CD45RA^−^CCR7^+^), effector memory CD8^+^ T cells (Tem, CD8^+^CD45RA^−^CCR7^−^), terminally differentiated effector memory CD8^+^ T cells (Tex, CD8^+^CD45RA^+^CCR7^−^). **CTV**: Cell Trace Violet. (For interpretation of the references to color in this figure legend, the reader is referred to the web version of this article.)

All patients underwent in-depth immunological assessments. At the time of initial evaluation, neutrophilia (P1), lymphopenia (P2, P5, P6), and eosinophilia (P1, P3) were recorded. Serum immunoglobulin levels showed low IgG and IgA in P1, panhypogammaglobulinemia in P2, high IgM in P3, low IgG and IgM in P5, and high IgE in P1 (Table 2). Detailed flow cytometric analysis, including T, B, NK, T and B cell subtypes, was performed in all patients at baseline and over time (Table 2). At baseline, reduced CD3^+^ T cell counts were observed in P2, P5, and P6. CD4^+^ T cells were low in P2, P3, P4, P5 and P6, while CD8^+^ T cells were decreased in P1, P2, P5, and P6. B and NK cell counts were overall normal, but moderately reduced B cell counts were detected in P1 and P2. Furthermore, P1 and P5 showed a low number of class-switched memory B cells. T cell subsets revealed markedly low percentages of naive CD4^+^ T cells and RTEs in all patients accompanied by high memory CD4^+^ T cells compared to healthy controls. Immunophenotyping of P1-P4 was further evaluated by tools developed to manage high-dimensional data (t-distributed stochastic neighbor embedding (t-SNE)), allowing a 2-dimensional visualization of clusters of phenotypically similar cells. t-SNE analysis demonstrated scarcely detected T cells when compared to healthy controls (Fig. 4A). Additionally, T cell subset analysis confirmed very low proportions of naïve CD4^+^ and CD8^+^ T cells and high effector memory CD4^+^ T cells (CD4^+^CD45RA^−^CCR7^−^) compared to healthy controls (Fig. 4B). The increase in effector memory CD4^+^ T cells was more prominent over time (Table 2). T cell activation measured by CD69 and CD25, and proliferation after anti-CD3/CD28 stimulation showed a reduction in P1-P4, which were more prominent in CD8^+^ T cells (Fig. 4C). Interestingly, CD4^+^ and CD8^+^ T cells of all patients exhibited different proliferative and activation capacities compared to healthy controls, delineating immunological heterogeneity among patients (Fig. 4C).

**Table 2:** The immunological evaluation of PAX1-deficient patients.

Parameters	P1 (Pre-HSCT)	P1 (Post-HSCT- 7 mo)	P1 (Post-HSCT-16 mo)	P2	P2	P2	P3	P3	P4	P4	P5	P5 (Post-TT-2 yrs)	P6	P6 (Post-HSCT-11 yrs)
Age at evaluation	3 mo	13 mo	21 mo	1 mo	12 mo	21 mo	6 mo	15 mo	6 yrs	11 yrs	2 mo	3 yrs	1 mo	12 yrs
Leukocytes; cell/μl	15,430	8540	9370	6320	6300	5450	14,200	5800	7650	6440	6000	5700	6670	5410
Neutrophils; cell/μl	9600 **(↑)**	5270	7380	4210	3000	2970	5500	2100	4340	4590	2500	2650	–	1820
Lymphocytes; cell/μl	3680	2650 **(↓)**	1310 **(↓)**	730 **(↓)**	2100 **(↓)**	1530 **(↓)**	6500	2900 **(↓)**	2650	1120 **(↓)**	2538 **(↓)**	1740	1400 **(↓)**	2860
Eosinophils; cell/μl	5740 **(↑)**	0 **(↓)**	60 **(↓)**	120	240	310	1160 **(↑)**	140	–	80	400	120	–	100
IgG (mg/dl)	194 **(↓)**	551 **(↓)**	644 **(↓)** **(On IVIG)**	255 **(↓)**	708 **(On IVIG)**	760 **(On IVIG)**	1570 **(On IVIG)**	1090 **(On IVIG)**	–	1258 **(On IVIG)**	325 **(↓)**	986 **(On SCIG)**	707	1490
lgA (mg/dl)	6 **(↓)**	19 **(↓)**	19 **(↓)**	11 **(↓)**	79	147	51	115	–	60 **(↓)**	25	33	<60	54 **(↓)**
IgM (mg/dl)	33	25 **(↓)**	35 **(↓)**	17 **(↓)**	27 **(↓)**	20 **(↓)**	229 **(↑)**	537 **(↑)**	–	55	26 **(↓)**	68	49	141
IgE (IU/ml)	5820 **(↑)**	10	506 **(↑)**	15	15	–	87	83	–	5.7	9	12	ND	–
CD3^+^ T cells (%)	67	35.5 **(↓)**	53	15 **(↓)**	35 **(↓)**	40 **(↓)**	31.1 **(↓)**	35.2 **(↓)**	73	71.5	5.4 **(↓)**	25.6 **(↓)**	0.1 **(↓)**	34.7 **(↓)**
CD3^+^ T cells (/mm^3^)	2465	940 **(↓)**	694 **(↓)**	109 **(↓)**	735 **(↓)**	612 **(↓)**	2021	1020 **(↓)**	1934	800 **(↓)**	139 **(↓)**	446 **(↓)**	0 **(↓)**	990 **(↓)**
CD4^+^ T cells (%)	47.6	12.8 **(↓)**	24.4 **(↓)**	13 **(↓)**	23.2 **(↓)**	16.5 **(↓)**	7.2 **(↓)**	10.5 **(↓)**	11.8 **(↓)**	20.2 **(↓)**	4.7 **(↓)**	18.9 **(↓)**	0 **(↓)**	27.9
CD4^+^ T cells (/mm^3^)	1751	339 **(↓)**	319 **(↓)**	94 **(↓)**	487 **(↓)**	252 **(↓)**	468 **(↓)**	295 **(↓)**	312 **(↓)**	226 **(↓)**	119 **(↓)**	329 **(↓)**	0 **(↓)**	800
CD8^+^ T cells (%)	2.4 **(↓)**	16.9	22.6	8 **(↓)**	7.9 **(↓)**	10.6 **(↓)**	20.6	25.4	45	41.8	0.5 **(↓)**	4.5 **(↓)**	0 **(↓)**	6.2 **(↓)**
CD8^+^ T cells (/mm^3^)	88 **(↓)**	447 **(↓)**	296 **(↓)**	58 **(↓)**	165 **(↓)**	162 **(↓)**	1339	736	1192	468	13 **(↓)**	80 **(↓)**	0 **(↓)**	180 **(↓)**
CD19^+^ B cells (%)	7 **(↓)**	37.7	28.5	13.8	39.8 **(↑)**	31.3 **(↑)**	39.9	35	16.8	21	64.9 **(↑)**	36.4 **(↑)**	55 **(↑)**	30.4 **(↑)**
CD19^+^ B cells (/mm^3^)	257 **(↓)**	999	373 **(↓)**	100 **(↓)**	835	479 **(↓)**	2593	1015	445	235	1648 **(↑)**	638	770	870 **(↑)**
CD16^+^56^+^ NK cells (%)	12.3	16.9	11.2	40 **(↑)**	15	21.5	16.2	20	8.8	1.1 **(↓)**	19.8	27.5	32 **(↑)**	34.3
CD16^+^56^+^ NK cells (/mm^3^)	452	447	146	292	315	328	1053	580	233	12 **(↓)**	503	483	450	980
CD3^+^ TCR α/β^+^ T cells (%)	97.9	68 **(↓)**	73.4 **(↓)**	ND	64.2 **(↓)**	43.6 **(↓)**	24.3 **(↓)**	ND	ND	70.6 **(↓)**	90.6	92.6	ND	–
CD3^+^ TCR γ/δ^+^ T cells (%)	1.5 **(↓)**	23 **(↑)**	21.1 **(↑)**	ND	31.7 **(↑)**	51.4 **(↑)**	68.7 **(↑)**	ND	ND	24.6 **(↑)**	6.4	7.3	ND	–
CD4^+^CD45RA^+^CD31^+^ T cells (%)	0.1 **(↓)**	10.8 **(↓)**	6.7 **(↓)**	ND	4.5 **(↓)**	4.3 **(↓)**	1.5 **(↓)**	1.7 **(↓)**	ND	2.1 **(↓)**	8.3 **(↓)**	9.3 **(↓)**	0 **(↓)**	1.7 **(↓)**
CD4^+^CD45RA^+^CCR7^+^ T cells (%)	0.2 **(↓)**	11.2 **(↓)**	8.9 **(↓)**	ND	10.3 **(↓)**	1.46 **(↓)**	0.2 **(↓)**	0.2 **(↓)**	ND	1.4 **(↓)**	10.8 **(↓)**	46.6	0 **(↓)**	4.3 **(↓)**
CD4^+^CD45RA^−^CCR7^+^ T cells (%)	0.7 **(↓)**	17	7.8 **(↓)**	ND	41.4 **(↑)**	8.5	4.4 **(↓)**	11.9	ND	14.7 **(↓)**	76.1 **(↑)**	21.1	0 **(↓)**	71.5 **(↑)**
CD4^+^CD45RA^−^CCR7^−^ T cells (%)	98 **(↑)**	66.9 **(↑)**	75.3 **(↑)**	ND	42.9 **(↑)**	79.3 **(↑)**	93.2 **(↑)**	85.8 **(↑)**	ND	81 **(↑)**	15	28.2 **(↑)**	0 **(↓)**	23.8
CD4^+^CD45RA^+^CCR7^−^ T cells (%)	1	4.7	7.9	ND	5.4	10.6	2.1	1.9	ND	2.8	0.4	4.1	0 **(↓)**	0.4
CD8^+^CD45RA^+^CCR7^+^ T cells (%)	0 **(↓)**	7.4 **(↓)**	7.7 **(↓)**	ND	19.8 **(↓)**	1.0 **(↓)**	1.5 **(↓)**	2.3 **(↓)**	ND	6.9 **(↓)**	34.4	65.6	0 **(↓)**	6.1 **(↓)**
CD8^+^CD45RA^−^CCR7^+^ T cells (%)	5.1	4.8	12.7 **(↑)**	ND	30.9 **(↑)**	1.0	0.1 **(↓)**	6.1	ND	3.8	54.6 **(↑)**	4.0	0 **(↓)**	64.8 **(↑)**
CD8^+^CD45RA^−^CCR7^−^ T cells (%)	92.9 **(↑)**	67.3 **(↑)**	67.8 **(↑)**	ND	24.9	52.3 **(↑)**	16.2	71.2 **(↑)**	ND	35	8.3	20.5	0 **(↓)**	16.9
CD8^+^CD45RA^+^CCR7^−^ T cells (%)	2 **(↓)**	20.4	11.7 **(↓)**	ND	24.3	45.5	82	20.2	ND	54.1	2.8 **(↓)**	10.0	0 **(↓)**	12.2
Naive B cells (%)	88.3	94.4	83.3	ND	73.8	76.6	89.3	ND	ND	89.3	97	91	ND	90.2
Naive B cells (/mm^3^)	226 **(↓)**	943	303 **(↓)**	ND	616	367 **(↓)**	2315	ND	ND	209	1599	581	ND	783 **(↑)**
NS memory B cells (%)	5.3	3.6	12.6	ND	6.8	5.7	3.3	ND	ND	2.9 **(↓)**	2	5.2	ND	4.7
NS memory B cells (/mm^3^)	14 **(↓)**	35	43	ND	56	27 **(↓)**	86	ND	ND	6 **(↓)**	33	33	ND	40
CS memory B cells (%)	1	0.6 **(↓)**	1.7 **(↓)**	ND	6.4	5.8	4.9	ND	ND	2.5 **(↓)**	0.2 **(↓)**	2.4	ND	1.5 **(↓)**
CS memory B cells (/mm^3^)	2.5 **(↓)**	5 **(↓)**	6 **(↓)**	ND	53	28	127	ND	ND	5 **(↓)**	3 **(↓)**	15	ND	12 **(↓)**

**Abbreviations:**CD4^+^ naive T cells (CD4^+^CD45RA^+^CCR7^+^), CD8^+^ naive T cells (CD8^+^CD45RA^+^CCR7^+^), recent thymic emigrants (CD4^+^CD45RA^+^CD31^+^), central memory CD4^+^ T cells (CD4^+^CD45RA^−^CCR7^+^), effector memory CD4^+^ T cells (CD4^+^CD45RA^−^CCR7^−^), terminally differentiated effector memory CD4^+^ T cells (CD4^+^CD45RA^+^CCR7^−^), central memory CD8^+^ T cells (CD8^+^CD45RA^−^CCR7^+^), effector memory CD8^+^ T cells (CD8^+^CD45RA^−^CCR7^−^), terminally differentiated effector memory CD8^+^ T cells (CD8^+^CD45RA^+^CCR7^−^), **NS B cell**: Non-switched memory B cells, **CS B cell**: Switched memory B cells. Abnormal values are shown with upper and lower arrows. For patient 6, naïve and memory phenotypes were determined using CD45RA/RO and CD27 instead of CCR7. **ND**: Not determined, **IVIG**: Intravenous immunoglobulin, **SCIG**: Subcutaneous immunoglobulin, **HSCT**: Hematopoietic stem cell transplantation, **TT**: Thymic transplantation.

**Fig. 5:**
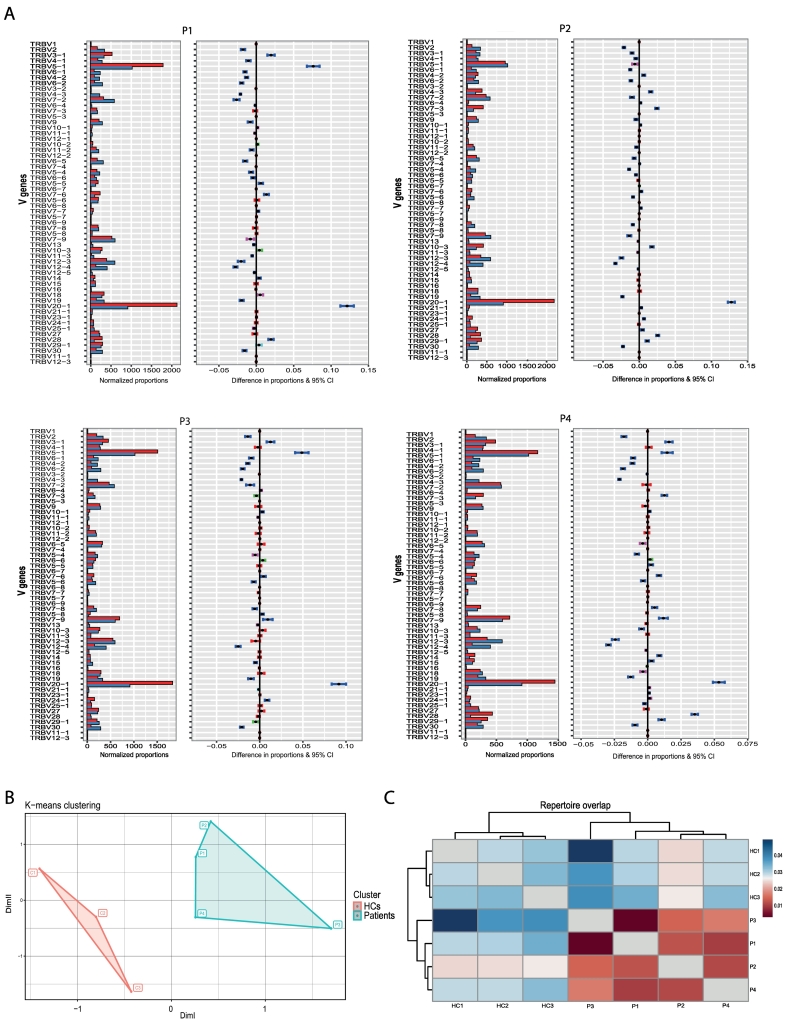
Skewed T cell receptor beta repertoire in PAX1 deficiency. **(A)** Individual V gene usage in patients compared to controls. Red bars represent the TRBV genes of the patients. Blue bars represent the TRBV genes of the healthy controls. Blue error bars indicate a significant difference in gene usage frequency compared to healthy controls (HCs). **(B)** Principle component analysis-based K-means clustering analysis of the V gene usage in the patients and healthy controls. V gene usage in CD4^+^ T cells of the patients and healthy controls. **(C)** Repertoire overlap analysis of clonotypes shared between patients and healthy controls. (For interpretation of the references to color in this figure legend, the reader is referred to the web version of this article.)

The Vβ repertoire was evaluated in CD4^+^ T cells of P1-P4, in CD8^+^ T cells of P3, and in CD3^+^ T cells from P5. In P1-P4, we first assessed the V gene usage in unique TRB clones. V gene usage frequency was skewed compared to three age-sex-matched healthy controls. The TRBV20–1 gene was the most skewed in the CD4^+^ TRB repertoire of P1-P4, followed by TRBV5–1 (Fig. 5A). A broader diversity was observed in V gene usage of healthy controls. Moreover, principle component analysis-based K-means clustering clearly showed the different CD4^+^ T cell V gene usage in the patients (Fig. 5B). Repertoire overlap measurement calculated by overlap coefficient analysis displayed distinct repertoire similarity between patients and healthy controls (Fig. 5C). We further observed restricted use of TRB V-J pairs in patients CD4^+^ T cells compared to healthy controls (Fig.E2). At the same time, there was a less restrictive pattern for CD8^+^ T cell V-J pairing in P3 (Fig.E3A). While a similar, most skewed TRBV20–1 gene in the CD8^+^ TRB repertoire of P3 was detected (Fig.E3B). The d50 diversity index demonstrated a restricted diversity for unique TCR beta V region (TRVB) sequences in P1-P4 (Fig.E3C, a and b). Another indicator of the low diversity was the top clonal proportions of the most abundant unique sequences. The most frequent 10, 100, and 1000 shared clones occupied a broader space in patients' CD4^+^ and CD8^+^ TRB repertoire compared to healthy controls (Fig.E3D, a and b). A different assay was used in P5 to study the Vβ repertoire before treatment with TT. TCRVβ spectratyping on isolated CD3^+^ T cells confirmed the presence of all Vβ families, yet with less than half showing a Gaussian distribution. Most families showed a skewed, sparse pattern with oligoclonal expansions (Fig.E4).

### 3.7 Normal *in vitro* T cell differentiation of patient-derived CD34^+^ cells

We co-cultured CD34^+^ cells from P3 and a healthy control with murine stromal cells in an ATO system in order to assess the *in vitro* differentiation potential into mature T cells [27]. Progressive maturation of T cell development was evaluated by determining the presence of cell surface markers expressed at different stages of T cell development (Fig. E5). After 6 weeks of co-culture, control and P3 CD34^+^ successfully differentiated into mature CD3^+^ TCRαβ^+^ cells, demonstrating a lack of hematopoietic cell-intrinsic defect in PAX1 deficiency.

### 3.8 Treatment and outcome

Four patients (P1, P3, P5, P6) were considered for corrective treatment. The other two patients (P2 and P4) were diagnosed with CID, and in light of dysgammaglobulinemia, they also received prophylactic antibiotics and were commenced on IgRT. Therapeutic management is detailed in the **Repository file**.

P1 and P6 received HSCT at 5 and 6 months of age for genetically undefined SCID, respectively. P1 underwent HSCT from a fully-matched sibling donor after receiving reduced intensity conditioning (RIC) with treosulfan and fludarabine. He achieved full donor chimerism (99%) one month after HSCT. The lymphocyte and T cell chimerisms decreased gradually and were no longer detectable at 8 months post-HSCT. After HSCT, he experienced Bacille Calmette Guerin (BCG)-itis but did not develop other complications such as graft *versus* host disease (GvHD) or Omenn Syndrome (Fig. 3B). He is now 21 months old and 16 months post-HSCT. Immunophenotyping shows low CD4^+^ and CD8^+^ T cells with persistent very low proportions of naïve T cells and RTEs (Table 2). He is being considered for a second procedure, ideally TT. P6 received a HSCT from a matched unrelated cord blood donor after RIC with treosulfan and fludarabine. Currently, she is 12 years old with 100% donor engraftment but remains T cell lymphopenic with poor thymic output (Table 2). She developed chronic skin GvHD but is otherwise clinically stable. She remains on azithromycin prophylaxis, but IgRT has been discontinued. P3 was referred for TT but died before this could be undertaken. P5 underwent TT at 11 months of age. He is now 3 years old with reconstituted T cell immunity (Table 2).

## 4 Discussion

This cohort includes a comprehensive evaluation of clinical and immunological characteristics of PAX1 deficiency not detailed in previously reported patients [[2], [3], [4], [5], [6]]. All patients but one harbor previously unidentified *PAX1* mutations. *In sillico* analysis revealed that the mutations could hamper the protein function by altering the integrity of both the PBD and IDR regions of PAX1. All pathogenic mutations showed loss-of-function nature with absent or reduced PAX1 transcriptional activity.

Although P2 had a milder clinical phenotype in this respect, ear, face, and skeletal anomalies were prominent in the other patients with structural anomalies of the vertebra and at the ends of the clavicle, which were evident in their chest X-rays. These abnormalities showed progression over time, as detected in P1, P2, and P6, indicating that regular screening during follow-up is required. All patients were diagnosed with immunodeficiency. Diminished T cells with markedly low percentages of naive CD4^+^ T cells and RTEs were consistent with defective thymic development. Furthermore, reduced T cell activation and proliferation were accompanied by restricted TCRVβ repertoires. Whilst P1 and P3 had the same mutation, one presented with an Omenn-like phenotype, while the other displayed classical SCID, illustrating the possible role of other environmental factors on clinical presentation. Furthermore, p.L70P variant of P2 showed some transcriptional activity compared to the other four variants tested. This confirms the hypomorphic nature of this PAX1 variant, which can explain the patient's milder overall clinical phenotype. However, P4, despite showing virtually absent transcriptional activity, also had a milder immunological phenotype consistent with variable penetrance of the immunodeficiency features even in the absence of transcriptional activity.

Beyond thymic aplasia/hypoplasia, we report a clinical overlap between DGS and PAX1 deficiency, including primary hypoparathyroidism and congenital heart defects. As with other disorders associated with DGS, we observe variable clinical penetrance. Notably, all patients except one experienced hypocalcemia, associated with low parathormone levels when measured. This was persistent in P1, P3, and P4. Primary hypoparathyroidism was not reported in the previous series of PAX1-deficient cases. This finding provides evidence that PAX1 has a role in the development of the parathyroid glands in humans. Syndromic patients with hypocalcemia and cardiac abnormalities should be evaluated for PAX1 deficiency. We also report intellectual disability in 4/6 patients, which is another variable clinical feature associated with DGS and OFCS1, suggesting early defects in embryogenesis contribute to this feature. We recommend early and regular neurodevelopment assessments to ensure appropriate supportive measures can be considered.

In this current report, we extensively evaluated the patients' immunological phenotypes. We show a lack of naïve CD4^+^ T cells and very abnormal TCR repertoires, in line with their immunodeficiency due to failure of thymus development, as previously reported [5]. This is further supported by the demonstration of the ability of PAX1-deficient CD34^+^ cells to differentiate into CD3^+^ TCRαβ^+^ cells *in vitro*. All patients' CD4^+^ and CD8^+^ T cells showed blunted proliferative and activation capacities compared to healthy controls, with CD8^+^ T cells more affected than CD4^+^ T cells. These were variable between patients, possibly indicating immunological heterogeneity in these responses. On the other hand, this variability can be due to the low T cell numbers used in the assay. It would be better to ensure that an equal number of control and patient T cells are stimulated under equivalent conditions, enabling a more accurate comparison of T cell proliferation.

One patient in our series developed an EBV-driven lymphoma. Patients with DGS can develop malignancies such as lymphoma, leukemia, neuroblastoma, hepatoblastoma, teratoid/rhabdoid tumor, and Wilms' tumor [33,34]. Even though the mechanisms of malignant transformation in DGS are not well-known, it may well be that the T cell deficiency and consequent persistent infections, such as EBV, are causally implicated, and this could also apply to the PAX1 deficiency [35]. Furthermore, one patient with a milder phenotype (P2) showed severe skewing to memory T cells, and most of the CD8^+^ T cells had a senescence phenotype (CD8^+^CD45RA^+^CCR7^−^), which can be associated with dysfunctional immunosurveillance [33]. Interestingly, the *PAX1* gene has been shown to suppress methylation in ovarian, cervical, oral, head and neck, and esophageal cancers and may act as a tumor suppressor gene [36,37]. All of these factors might contribute to a predisposition to the malignant disease in PAX1-deficient patients.

IgRT and antimicrobial prophylaxis can protect patients from recurrent infections, but definitive corrective therapy is required for those with severe immunodeficiency. Reporter assays and molecular modelling, as shown here, can help determine treatment if the need for corrective therapy is unclear. Athymic disorders, such as cDGS and Nude SCID (FOXN1), have been corrected with TT [38,39]. The mechanism involved in immunodeficiency suggests that TT should restore the immune phenotype of PAX1 deficiency. As reported recently, TT was used successfully in one patient from this series, P5 [40]. Two years post-TT, P5 was found to have 60% of naïve CD4^+^ T cells, corresponding to 378 cells/mm^3^. In contrast, P1 and P6 who received HSCT did not show any significant thymic output, with absolute naïve T cell counts at 16 months and 11 years post-HSCT remaining below 50 cells/mm^3^, the threshold routinely used to define complete athymia in patients with circulating T cells when considering eligibility for corrective treatment with TT [39]. Four previously reported PAX1-deficient patients underwent HSCT, but T cell immunity remained compromised, and naive T cells did not develop [5]. Overall this is in line with normal T cell differentiation of CD34^+^ cells from P3 in an ATO, suggesting that HSCT cannot robustly correct the T cell deficiency in this condition. As shown for cDGS, the long-term immunological function after HSCT remains significantly compromised [41,42]. However, the passive transfer of mature T cells during HSCT may confer some partial and relatively long-lived T cell immunity. This may have been the mechanism by which some improvement in immunity was achieved after HSCT in P6. In patients with significant viral infections, HSCT from a matched family donor might be considered to restore some immunity since the immune reconstitution after TT is very slow [38]. In other circumstances, TT should be recommended as the definitive treatment. HSCT from unrelated donors in this situation has very poor outcomes in athymic patients [41].

In summary, human PAX1 deficiency causes syndromic features and SCID/CID characterized by impaired T cell development and susceptibility to infections, autoimmunity, and malignancy, with variable clinical penetrance of the different features. Our study expands the spectrum of the disease and provides a detailed natural course by describing new features, including clinical heterogeneity and hypoparathyroidism overlapping with DGS. PAX1 deficiency should be considered in patients with syndromic features who suffer from infections, Omenn-like skin disease, chronic diarrhea, or hypocalcemia. Mutation modelling and reporter assays may help determine which patients require corrective treatment with TT.

## Author contributions

N.Y., A.Y.K. and S.B. conceptualized and supervised the study. H.K., T.K., M.B., O.M.D., M.C.C., B.E., S.A., Y.T.F., F.P., A.T., H.G., F.B.C., I.S.K., and M.E. performed the experiments. P.C. and H.G. performed and analyzed the modelling studies. N.Y., S.B., A.Y.K., H.K., R.B., A.P.S., S.B.E., M.Y.A., D.A., S.C., A.K., E.K.A., A.O., S.G., E.H., A.W., F.O., and E.G*.*D. provided patient care, collected samples and clinical data. N.Y., A.Y.K., L.D.N., E.G*.*D., and S.B. wrote the paper. All authors reviewed and approved the final version of the manuscript.

## Funding

This work was supported by grants from the 10.13039/501100004410Scientific and Technological Research Council of Turkey (318S202) and 10.13039/501100008386Marmara University Scientific Research Project Coordination Unit (ADT-2022–10661) to S.B., and in part, by 10.13039/501100001659Deutsche Forschungsgemeinschaft (DFG) through GRK 2158/2 (project number 270650915) to H.G.. TCR repertoire study was also supported by Scientific Research Projects Coordination Unit of 10.13039/501100005378Hacettepe University (THD-2022–20180) and the 10.13039/501100004410Scientific and Technological Research Council of Turkey (121S667) to B.E.. A.Y.K. is supported by the 10.13039/100010269Wellcome Trust (222096/Z/20/Z). E.G*.*D., E.H., and the UCL GOSH Thymus Transplantation program are supported by LetterOne in conjunction with GOSH Children's Charity. All research at GOSH is supported by the UK National Institute of Health Research and Great Ormond Street 10.13039/100014461Biomedical Research Centre. L.D.N. is supported by the Division of Intramural Research Program, 10.13039/100000060National Institute of Allergy and Infectious Diseases, NIH (AI001222).

## Declaration of Competing Interest

All authors declare no conflict of interest to disclose.

## Data Availability

Data will be made available on request.

## Appendix A Supplementary data

Supplementary material 1 Image 1

Supplementary material 2 Image 2

Supplementary material 3 Image 3

Supplementary material 4 Image 4

Supplementary material 5 Image 5

Supplementary material 6 Image 6

Supplementary material 7 Image 7
