# Corrigendum to “Expanding the clinical and immunological phenotypes of PAX1-deficient SCID and CID patients” [Clinical Immunology 255 (2023) 109757]

**DOI:** 10.1016/j.clim.2023.109799

**Published:** 2023-11

**Authors:** Nalan Yakici, Alexandra Y. Kreins, Mehmet Cihangir Catak, Royala Babayeva, Baran Erman, Heather Kenney, Hatice Eke-Gungor, Pablo A. Cea, Tomoki Kawai, Marita Bosticardo, Ottavia Maria Delmonte, Stuart Adams, Yu-Tong Fan, Francesca Pala, Ayberk Turkyilmaz, Evey Howley, Austen Worth, Hakan Kot, Asena Pinar Sefer, Altan Kara, Alper Bulutoglu, Sevgi Bilgic-Eltan, Melek Yorgun Altunbas, Feyza Bayram Catak, Ibrahim Serhat Karakus, Emrah Karatay, Sidem Didar Tekeoglu, Metin Eser, Davut Albayrak, Senol Citli, Ayca Kiykim, Elif Karakoc-Aydiner, Ahmet Ozen, Sujal Ghosh, Holger Gohlke, Fazil Orhan, Luigi D. Notarangelo, E. Graham Davies, Safa Baris

**Affiliations:** a Department of Pediatrics, Division of Pediatric Allergy and Immunology, Faculty of Medicine, Karadeniz Technical University Trabzon, Turkey; b Great Ormond Street Institute of Child Health, Infection, Immunity and Inflammation Research & Teaching Department, University College London, London, United Kingdom; c Department of Immunology and Gene therapy, Great Ormond Street Hospital for Children NHS Foundation Trust, London, United Kingdom; d SIHMDS-Hematology, Great Ormond Street Hospital for Children NHS Foundation Trust, London, United Kingdom; e Division of Pediatric Allergy and Immunology, School of Medicine, Marmara University, Istanbul, Turkey; f Istanbul Jeffrey Modell Diagnostic and Research Center for Primary Immunodeficiencies, Istanbul, Turkey; g The Isil Berat Barlan Center for Translational Medicine, Istanbul, Turkey; h Institute of Child Health, Hacettepe University, Ankara, Turkey; i Can Sucak, Research Laboratory for Translational Immunology, Center for Genomics and Rare Diseases, Hacettepe University, Ankara, Turkey; j Immune Deficiency Genetics Section, Laboratory of Clinical Immunology and Microbiology (LCIM), National Institute of Allergy and Infectious Diseases (NIAID), NIH, Bethesda, MD, USA; k Division of Pediatric Allergy and Immunology, Kayseri City Training and Research Hospital, University of Health Sciences, Kayseri, Turkey; l Institute for Pharmaceutical and Medicinal Chemistry, Heinrich Heine University, Düsseldorf, Germany; m Shizuoka Children's Hospital, Shizuoka, Department of Allergy and Clinical Immunology, Japan; n Department of Medical Genetics, Faculty of Medicine, Karadeniz Technical University Trabzon, Turkey; o TUBITAK Marmara Research Center, Gene Engineering and Biotechnology Institute, Gebze, Turkey; p School of Medicine, Marmara University, Istanbul, Turkey; q Department of Radiology, Marmara University Pendik Training and Research Hospital, Istanbul, Turkey; r Department of Pediatric Immunology, Hacettepe University, Ankara, Turkey; s Department of Medical Genetics, Umraniye Education and Research Hospital, University of Health Sciences, Istanbul, Turkey; t Department of Pediatrics, Division of Pediatric Hematology, Medicalpark Hospital, Samsun, Turkey; u Department of Medical Genetics, Faculty of Medicine, Recep Tayyip Erdogan University, Rize, Turkey; v Department of Pediatrics, Division of Pediatric Allergy and Immunology, Faculty of Medicine, Istanbul University-Cerrahpasa, Istanbul, Turkey; w Department for Pediatric Oncology, Hematology and Clinical Immunology, Medical Faculty, Center of Child and Adolescent Health, Heinrich Heine University, Düsseldorf, Germany; x Institute of Bio- and Geosciences (IBG-4: Bioinformatics), Forschungszentrum Jülich GmbH, Jülich, Germany

The authors regret to inform you that the name and affiliation of one author (K: Hatice Eke Gungor) was unintentionally written wrong.

The corrections should be as follows:

Name: Hatice Eke-Gungor.

Affiliation: ^k^Division of Pediatric Allergy and Immunology, Kayseri City Training and Research Hospital, University of Health Sciences, Kayseri, Turkey.

The authors would like to apologize for any inconvenience caused.

