# Supplementary material for: Expanding the clinical and immunological phenotypes of PAX1-deficient SCID and CID patients
Source: Clin Immunol. 2023 Oct;255:109757. doi: 10.1016/j.clim.2023.109757 (PMC10958138; doi:10.1016/j.clim.2023.109757)
Supplement: Supplementary file 1 — Supplementary material 1 [file mmc1.docx]

**Expanding the Clinical and Immunological Phenotypes of PAX1-deficient SCID and CID Patients**

**Yakici et al.**

**Supplementary File**

**Materials and methods**

**Antibodies and flow cytometry.** To determine deep lymphocyte subsets, the following monoclonal antibodies (mAbs) were used: Fluorescein isothiocyanate (FITC)-conjugated CD3 (UCHT1, 1:50, BC, FRA), Allophycocyanin (APC)-conjugated CD3 (33-2A3, 1:50, Immunostep, Spain), APC-Alexa Fluor 700 (APC-A700) CD4 (13B8.2, 1:50, BC), FITC CD4 (HP2/6, 1:50, Immunostep), APC CD4 (HP2/6, 1:50, Immunostep), Krome Orange (KO) CD45 (J33, 1:50, BC), Alexa Fluor 750 (APC-A750) CD45RA (2H4DH11LDB9, 1:50, BC), Phycoerythrin (PE) CD197 (CCR7) (G043H7, 1:50, BC), Pycoerythrin-Cyanin 7 (PC7) CD8 (SFCI21Thy2D3, 1:50, BC), APC-A700 CD14 (RMO52, 1:50, BC), PE CD16 (3G8, 1:50, BC), Pycoerythrin-Cyanin 5.5 (PC5.5) CD56 (N901, 1:50, BC), APC-A750 CD19 (J3-119, 1:50, BC), PB CD20 (B9E9, 1:50, BC), PB CD21 (BL13, 1:50, BC), PB  CD31 (5.6E, 1:50, BC), PC5.5 CD38 (LS198-4-3, 1:50, BC), Phycoerythrin-Texas Red-x (ECD) CD45RO (UCHL1, 1:50, BC), and FITC IgD (IA6-2, 1:50, BC). For lymphocyte subset analysis, 100µl of whole blood was incubated with mAbs against surface markers for 20 minutes in the dark at room temperature. Red cells were lysed and washed before acquisition. All stained cells were acquired with a Navios EX cytometer (Beckman Coulter) and analyzed with Kaluza Analysis Software (Version 2.1).

Peripheral T cells were divided into subpopulations as follows: CD4^+^ naive T cells (CD4^+^ CD45RA^+^ CCR7^+^), central memory CD4^+^ T cells (CD4^+^ CD45RA^–^ CCR7^+^), effector memory CD4^+^ T cells (CD4^+^ CD45RA^-^ CCR7^-^), terminally differentiated effector memory CD4^+^ T cells (TEMRA, CD4^+^ CD45RA^+^ CCR7^-^), CD8^+^ naive T cells (CD8^+^ CD45RA^+^ CCR7^+^), central memory CD8^+^ T cells (CD8^+^ CD45RA^–^ CCR7^+^), effector memory CD8^+^ T cells (CD8^+^ CD45RA^–^ CCR7^–^), and TEMRA CD8^+^ T cells (CD8^+^ CD45RA^+^ CCR7^–^). Peripheral B cells were classified into four distinct populations: naive mature B cells (CD19^+^ CD27^–^ IgD^+^), non-class-switched memory B cells (CD19^+^ CD27^+^ IgD^+^), class-switched memory B cells (CD19^+^ CD27^+^ IgD^-^) and autoreactive CD21^low^CD38^low^ B cells.

CD25 upregulation and proliferation assay were performed by isolation of peripheral blood mononuclear cells and stimulation in anti-CD3/anti-CD28 (1μg/ml each) 96-well plates for 3 days, following labeling with cell violet (Thermo Fisher) according to manufacturer’s instructions. CD69 upregulation was evaluated after 24 hours of stimulation with anti-CD3/anti-CD28. Stained cells were acquired by Navios EX cytometer (Beckman Coulter). Cytobank software (Beckman Coulter) was used to analyze the samples with Navios Ex cytometer.

**Genetic studies.** For Whole Exome Sequencing (WES), genomic DNA was extracted from peripheral blood samples, and 1$\text{μ}$g of DNA was used for exome capture using the IDT XGen exome target design or Agilent SureSelect Human All Exon. Generated libraries were sequenced using 75 bp paired-end sequencing on an Illumina NovaSeq-6000 and BGISeq-500 platform. Captured fragments were sequenced to achieve a minimum of 85% of the target bases covered at 20x or greater coverage. Sequence reads were mapped and aligned to the hg19 human genome reference assembly using Burrows-Wheeler Aligner (BWA)-mem and Single-Nucleotide Variants (SNV), and small Insertion/Deletion (INDEL) variants were called using Genome Analysis Toolkit (GATK). Variants were then annotated by VEP and ANNOVAR and filtered by GEMINI (GEnome MINIng) based on population allele frequency, potential genetic models, and variant deleterious predictions. In more detail, outputted variants were filtered by read depth (RD > 20), genotype quality score (GQ > 15), minor allele frequency (MAF < 0.01, as reference database 1K Genome and gnomAD employed), Combined Annotation Dependent Depletion (CADD) scores (CADD Phred > 5 or not available) and Variant Allele Frequency (VAF > 0.25). Along with these filters also, synonymous variants were filtered out. Finally, the possible effects of the variants on protein function were determined using in-silico pathogenicity prediction tools such as SIFT, Polyphen, and MutationTaster. Conservation of the mutated region was evaluated based on GERP scores. Clinical and laboratory phenotypes accompanied by the autosomal recessive inheritance model (due to the consanguinity in all families) were prioritized during the evaluation. The pathogenicity of all disease-attributable gene variants was re-evaluated using the updated guideline for interpretation of molecular sequencing by the American College of Medical Genetics and Genomics (ACMG) criteria ^1, 2^.

Sanger sequencing was performed to confirm the mutation identified by the WES. Briefly, genomic DNA was amplified by a polymerase chain reaction and amplimers were sequenced using the Big Dye Terminator v1.1 Cycle Sequencing Kit (Applied Biosystems; Life Technologies, Darmstadt, Germany) on an Applied Biosystems 3130 Genetic Analyzer.

**Structural analysis of PAX1 variants.** For structural analysis, two independent predictions were performed: one for the first helical bundle (residues 101 to 170) and another for the second helical bundle (residues 170 to 235). The two models were merged employing MODELLER ^3^, using the crystal structure of PAX6 (PDB PAX6) as a template. 100 models were generated. The best five were selected according to their Dopescore ^4^ and further evaluated using TopScore ^5^ to choose the best model. A low-quality region consisting of the last six residues at the C-terminus was further refined using GalaxyLoop refinement. To determine the co-evolutionary covariance of residue P162, we constructed a multiple sequence alignment using Jackhmmer ^6^ setting an E-value cut-off of 10^-6^ and performing eight iterations. The pairwise co-evolutionary couplings were determined using GREMLIN ^7^. Residue statistics for the amino acid distribution at position 162 of homologous PAX1 sequences were calculated using BioPython and Pandas and visualized with Matplotlib in Python 3.8.3.

**Luciferase reporter assay for analysis of Pax1 transcriptional activity**. Cells were co-transfected with 30ng of either wild type (WT) or mutant *Pax1* expression plasmids, 15ng firefly reporter plasmid *Nkx3-2*-pGL4.10luc2, and 3ng of pRL-TK vector (Promega) for normalization. After 48 hours, cell lysates were collected and firefly and renilla luciferase activities were measured using a Dual-Luciferase Reporter Assay kit (Promega) and Filter-based multi-mode microplate reader, FLUOstar Omega (BMG Labtech). To correct for variation in transfection efficiency, firefly luciferase activity was normalized to renilla luciferase activity. The empty pCMV-HA-N vector was assumed to have 0% luciferase activity, whereas the Pax1^WT^ vector was considered to have 100% luciferase activity.

**T cell receptor beta (TRB) repertoire analyses.** Repertoire libraries were prepared by SMARTer® Human T-cell receptor (TCR) α/β Profiling Kit (Takara). Using SMART technology (Switching Mechanism at 5′ End of RNA Template) and a 5′ RACE-like approach, complete V(D)J variable regions of TCR transcripts were captured. It also includes unique molecular identifiers for PCR error correction and clonotype quantification during data analysis. Libraries were sequenced with paired-end, 2x300 base pair reads using Miseq next-generation sequencing (NGS) system (Illumina). Sequence data was analyzed for sequencing error correction, clonotype calling, and mapping statistics by Cogent NGS Immune Profiler (Takara). Repertoire diversity and gene usage analyses were done by Immunarch and IMGT/HighV-QUEST software.

***In vitro* T cell differentiation study**. CD34^+^ peripheral blood cells from a normal donor and patient P3 were positively selected using the CD34 MicroBead kit UltraPure (Miltenyi Biotech) on the AutoMACS Pro Separator. 1000-1500 CD34^+^ cells were combined with 150,000 MS5-hDLL4 cells per ATO. Each ATO (5 µl) was then plated in a 0.4 µM Millicell Transwell insert, placed on a well of a 6-well plate containing 1 ml of complete RB27 medium supplemented with rhIL-7 (5 ng/ml), rhFlt3-L (5 ng/ml) and 30 µM l-ascorbic acid 2-phosphate sesquimagnesium salt hydrate. Each insert contained a maximum of two ATOs. For the first 3 weeks of culture, the medium was supplemented with 10 ng/ml of rhSCF. After 5 weeks in culture, MACS buffer (PBS with 0.5% BSA and 2 mM EDTA) was added to each well, and ATOs were dissociated by manual pipetting. Cells were then pelleted, resuspended in FACS Buffer (PBS with 2% FBS), counted, and stained with the following antibodies: CD5 PE-Cy7 (cl. UCHT2, eBioscience), CD7 Alexa Fluor 700 (cl. eBio124-1D1, eBioscience), TCRαβ PerCP-Cy5.5 (cl. IP26, Biolegend), CD4 APC-Cy7 (cl. SK3, BD Biosciences), CD56 FITC (cl. MEM-188, Biolegend), CD1a APC (cl. HI149, Biolegend), TCRγδ FITC (cl. B1, Biolegend), CD45 V500 (cl. HI30, BD Biosciences), CD3 BV421 (cl. UCHT1, BD Biosciences), CD8β PE (cl. 2ST8.5H7, BD Biosciences), LIVE/DEAD™ Fixable Yellow Dead Cell Stain Kit (Thermo Fischer). Events were acquired on a BD LSR II Fortessa (BD Biosciences, San Jose, CA) and analyzed using FlowJo software version 10.6.1 (FlowJo, LLC, Ashland, OR).

**Detailed summary of patients (P1-P6)**

P1 was a boy, born full-term to consanguineous parents after an uneventful pregnancy, except for a mildly symptomatic COVID-19 infection in the 4^th^ month, which did not require any medical interventions. At 15 days old, he suffered from rapidly worsening dermatitis covering his whole body. He also showed chronic diarrhea within the following month without a pathogen detected. At 1.5 months of age, he was admitted with chronic diarrhea and found hypocalcemic (6.1 mg/dl) with normal but inappropriate lower parathyroid hormone level (39.6 ng/L; normal level: 12-88 ng/L) and normal vitamin D and serum magnesium levels. He was diagnosed with primary hypoparathyroidism and commenced on calcium supplementation. On physical examination, he had a diffuse erythematous skin rash and dysmorphic features, including bilateral microtia, nasal root flattening, hypertelorism, epicanthus, micrognathia (more prominent on the right side), and undescended testicles. Cranial magnetic resonance imaging (MRI) showed bilateral agenesis of external auditory canals with normal inner ear structures. Bilateral hearing loss was detected. Further imaging showed split cervical vertebral bodies and hooked distal clavicles with no thymus shadow. High serum IgE and eosinophilia were seen, and his immunophenotyping analysis showed normal absolute T lymphocyte counts but with negligible proportions of CD45RA^+^CCR7^+^ naïve T cells and CD45RA^+^CD31^+^ recent thymic emigrants (RTEs). Mild B cell lymphopenia was noted, together with hypogammaglobulinemia. Maternal chimerism was excluded. 22q11.2 deletion syndrome was excluded by FISH analysis. Overall, these results were compatible with a T^low^B^+^NK^+^ SCID phenotype, initially remaining genetically undefined. The patient had previously received Bacille Calmette Guerin (BCG) vaccination and was therefore commenced on anti-BCG prophylaxis with isoniazid and rifampicin. He had diffuse lung infiltration on thoracic computed tomography (CT), which regressed with long-term antibiotic therapy. At 5 months, he underwent hematopoietic stem cell transplantation (HSCT) from a fully-matched sibling donor after receiving reduced intensity conditioning (RIC) with treosulfan and fludarabine. He achieved full donor chimerism (99%) one month after HSCT. Three weeks post-HSCT, he developed BCG lymphadenitis. Despite treatment with isoniazid, rifampicin, and pyrazinamide, the lymphadenitis progressed. Therefore, it was decided to discontinue the immunosuppressive treatment at 2 months post-HSCT, and lymphadenitis resolved with 9 months of anti-BCG therapy. The chimerism decreased gradually, and at the 5^th^ month of transplantation, donor chimerism was detected at 13%. In the meantime, there were no clinical findings of Omenn syndrome. In the 8^th^ month of transplantation, he was non-chimeric and his immunological analysis demonstrated low CD4^+^ and CD8^+^ T cells with persistent very low proportions of naïve T cells and RTEs. Currently, he is 16 months post-HSCT with no donor T cell engraftment and absent immune reconstitution. He remains clinically stable on trimethoprim-sulfamethoxazole prophylaxis and immunoglobulin replacement therapy (IgRT). At 18^th^ months, he underwent a successful orchiopexy surgery. He is now 21 months old and continues to use an external hearing device and is still on calcium supplementation in light of inadequate PTH activity, achieving normal serum calcium levels.

P2 is the first child of non-consanguineous parents. After delivery, he was admitted to the neonatal intensive care unit due to respiratory distress, hypoglycemia, and hypocalcemic seizures. His laboratory results showed hypocalcemia (7.2 mg/dl), and hyperphosphatemia (7.4 mg/dl) with a normal but insufficient increase in parathyroid hormone level (22 ng/L; normal level: 12-88 ng/L), led to diagnosing hypoparathyroidism. Furthermore, newborn screening revealed congenital hypothyroidism. Therefore, he was commenced on L-thyroxine. On physical examination, mild low-set ears, broad forehead, short nose with a low nasal bridge, hypertelorism, epicanthus, micrognathia, long philtrum, and thin upper lip were noted. He demonstrated persistent lymphopenia together with hypogammaglobulinemia and mild eosinophilia. Immunophenotyping showed isolated T cell lymphopenia compatible with a T^low^B^+^NK^+^ CID phenotype**.** He had a normal karyotype and 22q11.2 deletion syndrome was ruled out by FISH analysis. His hearing test was normal. The echocardiogram showed a small secundum atrial septal defect. A skeletal survey showed nearly normal findings at the first evaluation but demonstrated progression during the follow-up and appeared to have hypoplastic vertebrae and scoliosis at 23 months old.

Mild neutropenia emerged at 2 months of age and resolved spontaneously at 8 months old, which was thought to be autoimmune induced by multiple drug usage. He is now 23 months old, clinically stable, and infection-free on trimethoprim-sulfamethoxazole prophylaxis and IgRT. The hypocalcemia improved in 2^nd^ month of life, and his last PTH level was normal (48.8 ng/L). However, he remains T cell lymphopenic with absent thymic output over time, and his lymphocyte numbers surge between 1000 and 2100/mm^3^ **(Table 2)**.

P3 was born at term to 2^nd^-degree related parents. He was admitted to the neonatal intensive care unit for 14 days at birth because of respiratory distress. He was re-admitted at 6 months of age due to pneumonia, EBV infection, and axillary lymphadenopathy. Family history was remarkable for multiple members with ear abnormalities and hearing loss, including one older sibling who died at 18 months due to severe pneumonia and secondary heart failure. Initial immunological evaluation at 6 months showed normal lymphocyte counts and mild eosinophilia. Immunophenotyping exhibited isolated T cell lymphopenia (T^low^B^+^NK^+^). Other immunological assessments are presented in **Table 2**. Having received BCG vaccination, a needle aspiration biopsy was performed on the left axillary lymph nodes, and staining with culture was negative for mycobacteria. He was also found to have a low blood CMV copy number (440 copies/mL), and treatment was initiated with ganciclovir. Concomitantly, he also developed transaminitis, autoimmune hemolytic anemia (AIHA), and mild thrombocytopenia. He was therefore commenced on high-dose intravenous immunoglobulin. The CMV viral load became undetectable, and the cytopenias resolved. He was hospitalized for bronchiolitis at 12 months of age with no specific pathogen detection. During the evaluation, he was found to be hypocalcemic without symptoms and was diagnosed with primary hypoparathyroidism, requiring calcium supplementation and calcitriol treatment (PTH: 10 ng/L, Ca: 7.9 mg/dL). On physical examination, he had micro and retrognathia, maxillary hypoplasia, telecanthus, bilateral epicanthus, small and anteverted nostrils, microtia, low set ears, down slanting palpebral fissures, and thin upper lip. No thymic shadow was seen on the chest X-ray, and skeletal anomalies were found, including C6 and C7 butterfly vertebrae and hooked distal clavicles. Temporal bone CT showed bilateral external auditory canal atresia. Thoracic CT showed multiple mediastinal enlarged lymph nodes and ground glass in the upper lobes of both lungs. The echocardiogram revealed a small secundum atrial septal defect. While receiving broad-spectrum antibiotics for pneumonia, including voriconazole for *Candida parapsilosis*, his lung involvement deteriorated, and new consolidation areas causing total loss of aeration in the upper right lung were observed. In addition, multiple nodules with irregular borders were noted in both lungs, some of them also showing cavitation. Pathological examination of the resected lesion showed EBV-positive diffuse large cell lymphoma. Suddenly, after the diagnosis, he developed a generalized tonic-clonic convulsion. A brain MRI showed an intracerebellar left hemispheric lesion with enlarged lateral ventricles, suspected to be a metastatic mass, which led to his death at 21 months old.

P4 was born to consanguineous parents at term. At birth, syndromic features were observed and became more prominent over time, including facial dysmorphism, hypertelorism, flattened nasal root, epicanthus, flat philtrum, long eyelashes, right facial paralysis, bilateral microtia and low-set ears, periauricular pits, short neck, prominent gibbus, hooked distal clavicles and cryptorchids. He had one term sibling without syndromic features who died within 24 hours after birth due to feeding aspiration. At 2 months, BCG vaccination was applied without side effects. He had one sibling with no syndromic features who died suddenly at 2 months. At 12 months of age, he was hospitalized for coombs positive AIHA. He was hypocalcemic and was diagnosed with primary hypoparathyroidism requiring calcium supplementation and calcitriol (PTH: 5 ng/L, Ca: 8 mg/dL). At 6-year-old he was referred for investigation of suspected immunodeficiency due to his syndromic features revealing T cell lymphopenia and hypogammaglobulinemia. He was diagnosed with CID, and antimicrobial prophylaxis (trimethoprim-sulfamethoxazole, fluconazole prophylaxis, and IgRT) was initiated. He is now 12 years old and remains on these therapies.

P5 was born at term to consanguineous parents with a history of multiple miscarriages by emergency cesarean due to a pathologic antepartum cardiotocography. He required respiratory support for the first five days of his life. The newborn screening later identified the patient as having absent T cell receptor excision circles. Immunophenotyping was compatible with a diagnosis of T^low^B^+^NK^+^ SCID, and subsequent whole exome sequencing detected a homozygous *PAX1* mutation. On clinical examination, syndromic features consistent with otofaciocervical syndrome were observed, including a flat nasal bridge, hypertelorism, low-set ears with preauricular pits, overfolded ears, and a hypoplastic mandible with retrognathia. In addition, the patient had a nevus flammeus birthmark on the nasal bridge and in the lumbosacral region. Subsequent skeletal imaging in the first year of life showed an aplastic left external auditory meatus. Bilaterally, the stapes were not attached to the oval window. Cochlea and semicircular canals showed increased sclerosis. Brainstem evoked response audiometry confirmed bilateral hearing loss. Imaging of the spine revealed increased sclerosis of the posterior parts of the vertebrae. At the same time, anterior portions of the lower thoracic and lumbar vertebral bodies were hypoplastic and scalloping leading to hyperkyphosis of the thoracic / hypolordosis of the lumbar spine and further scoliosis. There were no problems with parathormone or calcium levels at any time point.

Ultrasound could not detect any thymic tissue. Initial immunology showed marked T cell lymphopenia. The patient was started on cotrimoxazole and fluconazole prophylaxis, and IgRT. In his first year, he had no significant infections; however, the patient developed scabies, which resolved with oral ivermectin. At 11 months of age, he underwent thymus transplantation after referral. He is now 3 years old with reconstituted T cell immunity but remains on IgRT. Management of non-immunological manifestations such as speech delay due to hearing loss and orthopedic issues are further challenges in this patient.

P6 was reported recently ^8^. She is included for more detailed clinical and immunological features, which were not fully described in that report. She was born to second-degree related parents. Their first child presented with a severe erythematous rash and died at three months due to severe RSV disease. Retrospectively a diagnosis of Omenn Syndrome was suspected in the context of genetically undefined SCID. Due to the significant family history, she was assessed at birth. She displayed syndromic features, including low set ears with right preauricular pit, left microtia and bilateral dysplastic pinna, hypertelorism, triangular facies with short palpebral fissures, flat nasal bridge and beak-like nose with hypoplastic *alae nasi*. Bilateral conductive hearing impairment was confirmed. A skeletal survey showed anteriorly hooked clavicles with broad distal ends, scoliosis, kyphosis, and hypoplastic cervical vertebrae, including several butterfly vertebrae. In the early neonatal period, she suffered from recurrent infections requiring antibiotics. She also had a history of neonatal tetany and was repeatedly found to be hypocalcemic, requiring calcium supplementation. A low PTH level (0.7 pmol/L, normal range: 2.0 to 6.8 pmol/L) was measured and alphacalcidol treatment was started. Hypocalcemia resolved spontaneously over time, and calcium supplementation was discontinued. Several years later, she again displayed tetany with low calcium levels and low PTH levels. The calcium supplementation was re-initiated.

Immunophenotyping showed no T cells, and she was diagnosed with T^-^B^+^NK^+^ SCID **(Table 2)**. A comparative genomic hybridization array did not detect a microdeletion of chromosome 22q11.2, and the genetic testing panel did not show variations in any known SCID genes. On MRI, a small homogeneous tissue was seen within the anterior mediastinum in keeping with a small thymus. At 6 months old, she received a matched unrelated cord blood HSCT after RIC with treosulfan and fludarabine. Currently, she is 12 years old with 100% donor engraftment but remains T cell lymphopenic with poor thymic output. She suffers from chronic skin graft-versus-host disease but is otherwise clinically stable and takes azithromycin prophylaxis. IgRT has successfully been discontinued. Abnormal rib cage and worsening thoracolumbar hyperkyphosis and lumbar hyperlordosis have been noted since infancy.

**Supplementary Figure and Table Legends**

**Figure E1. Variants of PAX1-deficient patients are located in a conserved area. (A)** Mapping of the mutations found in patients 1-6 on the PAX1 sequence. The upper plot shows the boundaries (peaks) between the domains that compose *PAX1*. The lower plot shows the predicted secondary structure elements along the PAX1 sequence (purple: α-helix, yellow: β-strand, cyan: coil or unstructured). The only structured region of the protein, the paired box domain, is highlighted in pale yellow. The position of each variant is highlighted with a red label and a red dashed line. (**B**) Evolutionary conservation of the mutated sites among different species. The localization of the variants is indicated by red color.

**Figure E2. Restricted TRB V-J pairs in PAX1 deficiency**. Heat map graphs of TRB V-J pairs in CD4^+^ T cells of the patients and 2 healthy controls (HCs). The analysis of P1 was conducted before HSCT.

**Figure E3. Decreased TRB V-J pairs in CD8^+^ T cells of PAX1 deficiency. (A)** V-J pairing of the CD8^+^ TRB repertoire of P3 in comparison with the healthy control (HC). **(B)** Individual V gene usage of CD8^+^ T cells of P3. Red bars represent the TRBV genes of the patient. Blue bars represent the TRBV genes of the healthy control. Blue error bars indicate significant differences in the gene usage frequency. **(C)** d50 diversity index of the patients’ TRB repertoire compared to healthy controls (HCs): **a**) Diversity of CD4^+^ T-cell repertoire of all patients. **b**) Diversity of CD8^+^ T-cell repertoire of P3. **(D)** The top clonal proportions of the most abundant unique sequences are characterized by 1-10, 11-100, and 101-1000 shared clones, occupying a broader space in the patients. The "clonal proportions" refers to the relative abundance of different clones of identical sequences within a sample. The selected number of clones with the highest frequencies (10, 100, and 1000) covered a wide range or occupied a larger proportion of the overall sequence diversity within the sample**: a**) Proportions of CD4^+^ TRB repertoire of all patients. **b**. Proportions of CD8^+^ TRB repertoire of P3.

**Figure E4.** TCRVβ spectratyping shows skewed and sparse patterns with oligoclonal expansions. The result of P5 on isolated CD3^+^ T cells is shown. All Vβ families are represented, but only 7/24 show a Gaussian distribution.

**Figure E5. Normal T cell differentiation in PAX1 deficiency.** *In vitro* T-cell differentiation of positively selected peripheral blood CD34^+^ cells obtained from a healthy control (Ctrl) and a subject carrying the *PAX1* variant (P3) after 6 weeks of culture in the ATO system. The FACS plots show expression of early and late T-cell differentiation markers CD7, CD5, CD1a, CD4, CD8b, TCR^a/b^, and CD3 upon gating on LIVE/DEAD (L/D)^–^ CD45^+^ CD56^–^ cells.

**Supplementary References**

1. Li Q, Wang K. InterVar: Clinical Interpretation of Genetic Variants by the 2015 ACMG-AMP Guidelines. Am J Hum Genet 2017; 100:267-80.

2. Richards S, Aziz N, Bale S, Bick D, Das S, Gastier-Foster J, et al. Standards and guidelines for the interpretation of sequence variants: a joint consensus recommendation of the American College of Medical Genetics and Genomics and the Association for Molecular Pathology. Genet Med 2015; 17:405-24.

3. Sali A. Comparative protein modeling by satisfaction of spatial restraints. Mol Med Today 1995; 1:270-7.

4. Shen MY, Sali A. Statistical potential for assessment and prediction of protein structures. Protein Sci 2006; 15:2507-24.

5. Mulnaes D, Gohlke H. TopScore: Using Deep Neural Networks and Large Diverse Data Sets for Accurate Protein Model Quality Assessment. J Chem Theory Comput 2018; 14:6117-26.

6. Johnson LS, Eddy SR, Portugaly E. Hidden Markov model speed heuristic and iterative HMM search procedure. BMC Bioinformatics 2010; 11:431.

7. Ovchinnikov S, Kamisetty H, Baker D. Robust and accurate prediction of residue-residue interactions across protein interfaces using evolutionary information. Elife 2014; 3:e02030.

8. Sherlaw-Sturrock C, Austin T, Baptista J, Gilmour K, Naik S. Dysmorphism and immunodeficiency - One of the differential diagnoses is PAX1 related otofaciocervical syndrome type 2. Eur J Med Genet 2022; 65:104523.
