# Supplementary material for: Expanding the clinical and immunological phenotypes of PAX1-deficient SCID and CID patients
Source: Clin Immunol. 2023 Oct;255:109757. doi: 10.1016/j.clim.2023.109757 (PMC10958138; doi:10.1016/j.clim.2023.109757)
Supplement: Supplementary file 2 — Supplementary material 2 [file mmc2.docx]

| **Patient** | **Transcript** | **Nucleotide change (human)** | **Amino acid change (human)** | **Nucleotide change (mouse)** | **Amino acid change (mouse)** | **Allele frequency (GnomAD)** | **CADD Score** |
| --- | --- | --- | --- | --- | --- | --- | --- |
| P1 and P3 | ENST00000398485.6 | c. 872C>A | p. S291* | c.841C>A | p. S281X | NA | 39.0 |
| P2-Maternal | ENST00000398485.6 | c.1541G>A | p. W514* | N/A | N/A | 0.00000415 | 35.0 |
| P2-Paternal | ENST00000398485.6 | c. 209T>C | p. L70P | c.171T>C | p. L64P | 0.000086 | 24.40 |
| P4 | ENST00000398485.6 | c.1005dupC | p. A336fs*15 | c. 981dupC | p. A327fs*15 | NA | NA |
| P5 | ENST00000398485.6 | c. 485C>T | p. P162L | c. 458C>T | p. P153L | NA | 27.9 |
| P6 | ENST00000398485.6 | c. 501del | p. S168A | c. 474del | p. S159A | NA | NA |

**Table E1.** The *PAX1* mutations with allele frequencies and CADD scores

**Abbreviation:** CADD: Combined annotation dependent depletion; NA: Not available.
