# Supplementary material for: Expanding the clinical and immunological phenotypes of PAX1-deficient SCID and CID patients
Source: Clin Immunol. 2023 Oct;255:109757. doi: 10.1016/j.clim.2023.109757 (PMC10958138; doi:10.1016/j.clim.2023.109757)
Supplement: Supplementary file 3 — Supplementary material 3 [file mmc3.pdf]

Figure E1

A

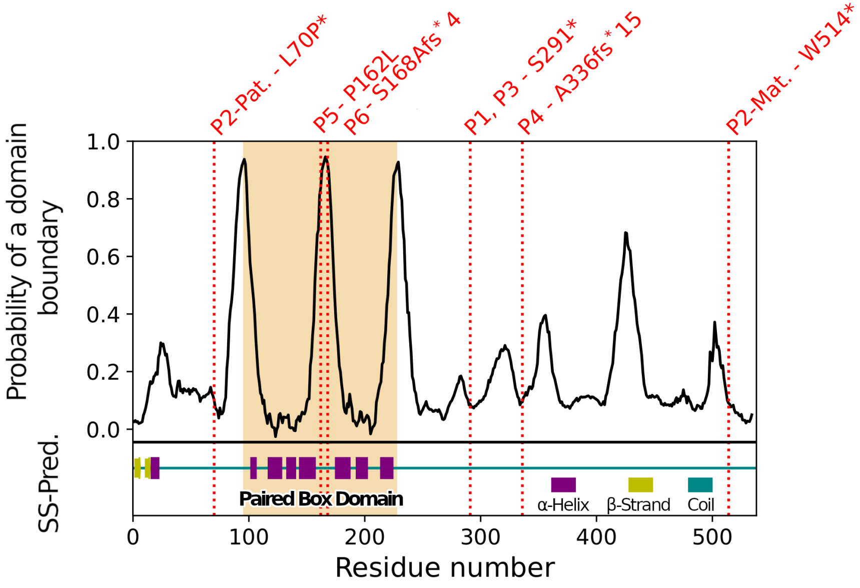
P2-Pat. - L70P*
P5- P162L
P6- S168Afs*4
P1, P3 - S291*
P4 - A336fs*15
P2-Mat. - W514*
Probability of a domain boundary
SS-Pred.
Paired Box Domain
α-Helix
β-Strand
Coil
Residue number
1.0
0.8
0.6
0.4
0.2
0.0
0
100
200
300
400
500

B

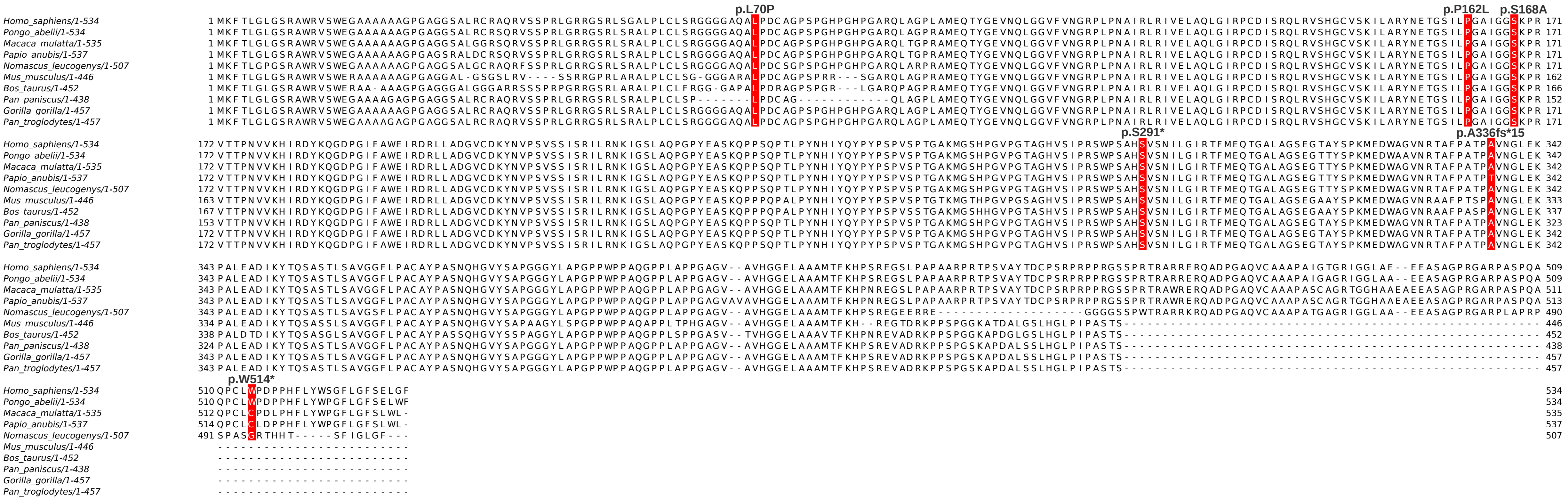
p.L70P
p.P162L
p.S168A
p.S291*
p.A336fs*15
p.W514*
Homo_saphiens/1-534
Pongo_abelii/1-534
Macaca_mulatta/1-535
Papio_anubis/1-537
Nomascus_leucogenys/1-507
Mus_musculus/1-446
Bos_taurus/1-452
Pan_paniscus/1-438
Gorilla_gorilla/1-457
Pan_troglodytes/1-457
