# Supplementary figures and images for: Expanding the clinical and immunological phenotypes of PAX1-deficient SCID and CID patients
Source: Clin Immunol. 2023 Oct;255:109757. doi: 10.1016/j.clim.2023.109757 (PMC10958138; doi:10.1016/j.clim.2023.109757)

## Figure E2

P1

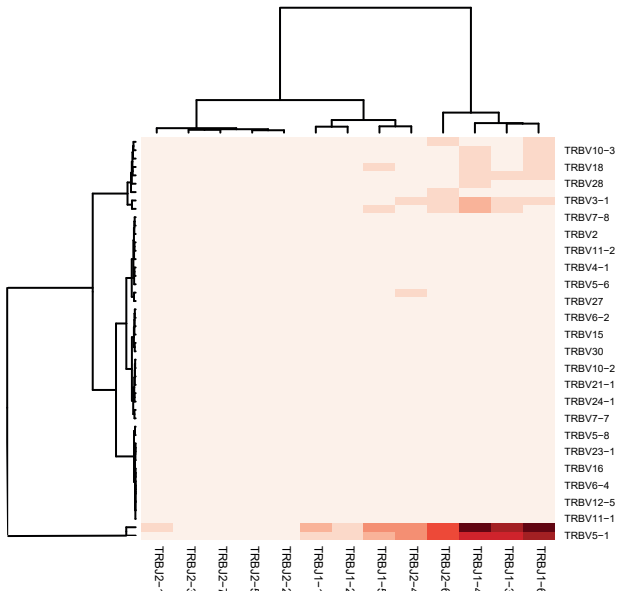

P2

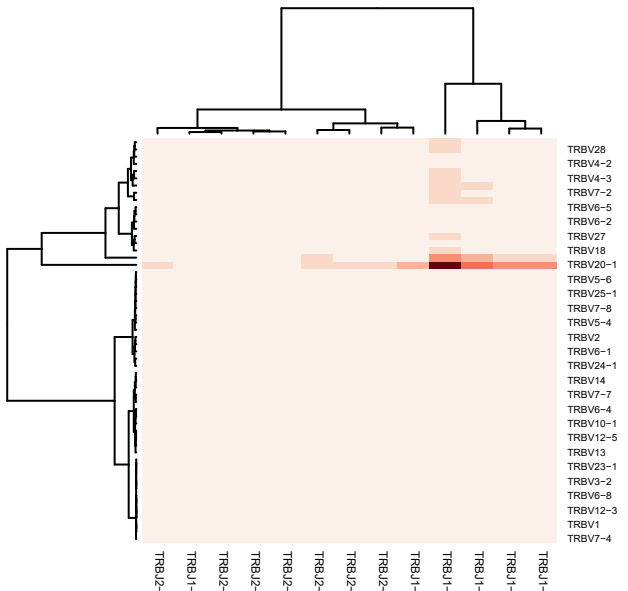

P3

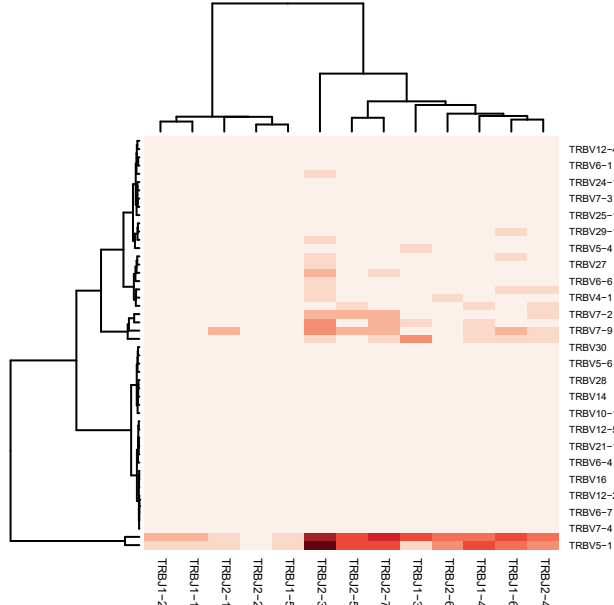

P4

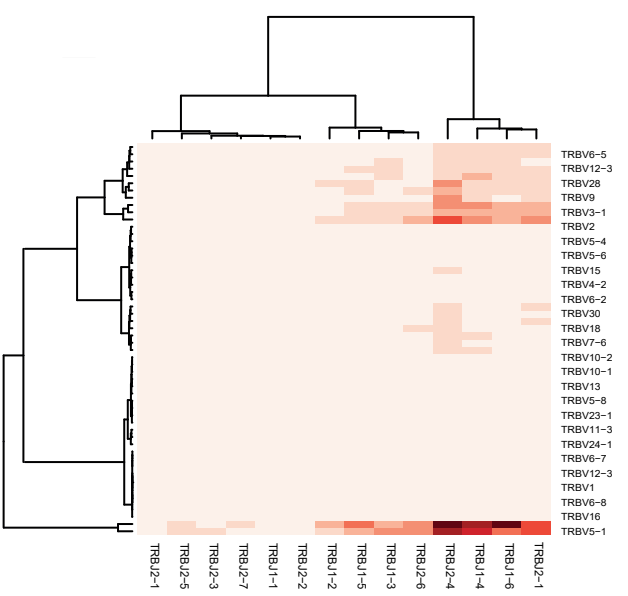

He

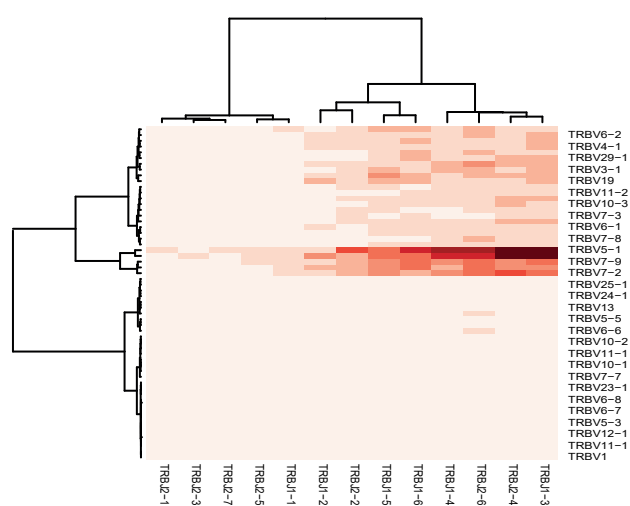

He

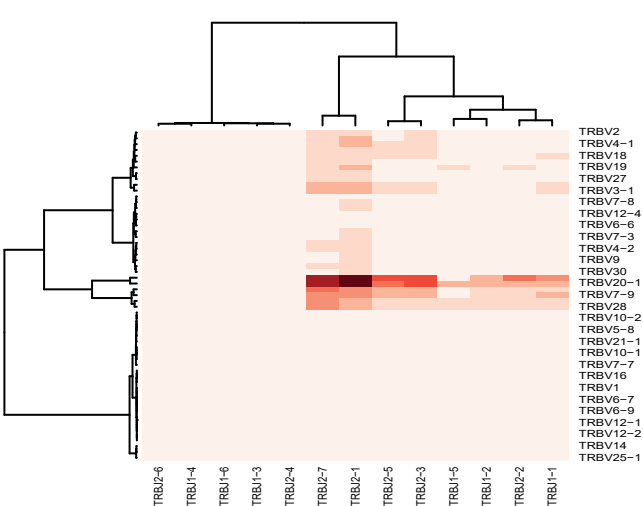

Supplement: Supplementary file 4 — Supplementary material 4 [file mmc4.pdf]

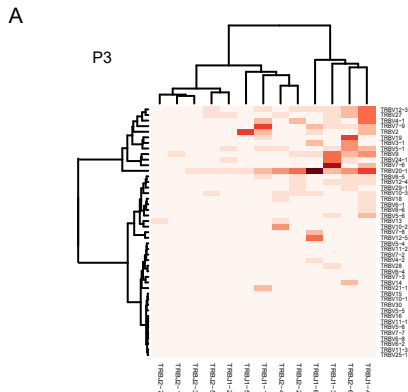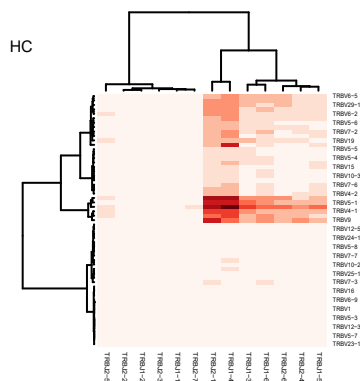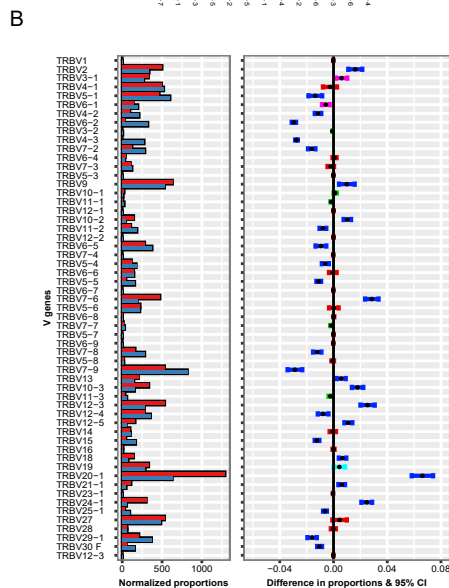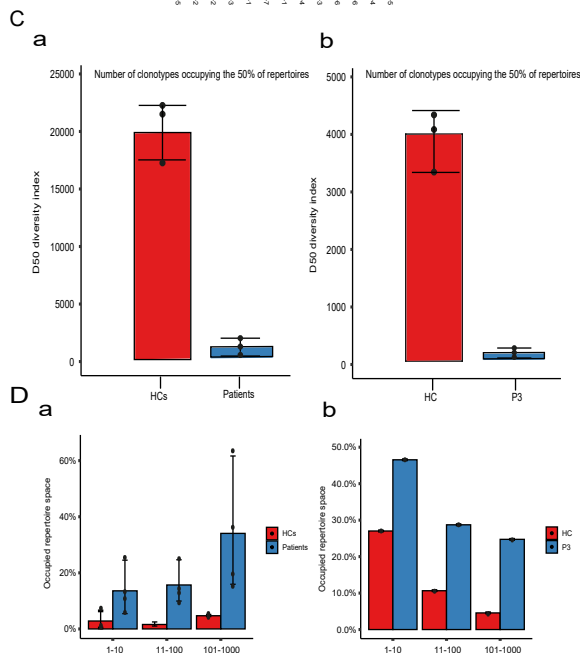

**Figure E3**

Supplement: Supplementary file 5 — Supplementary material 5 [file mmc5.pdf]

Figure E4

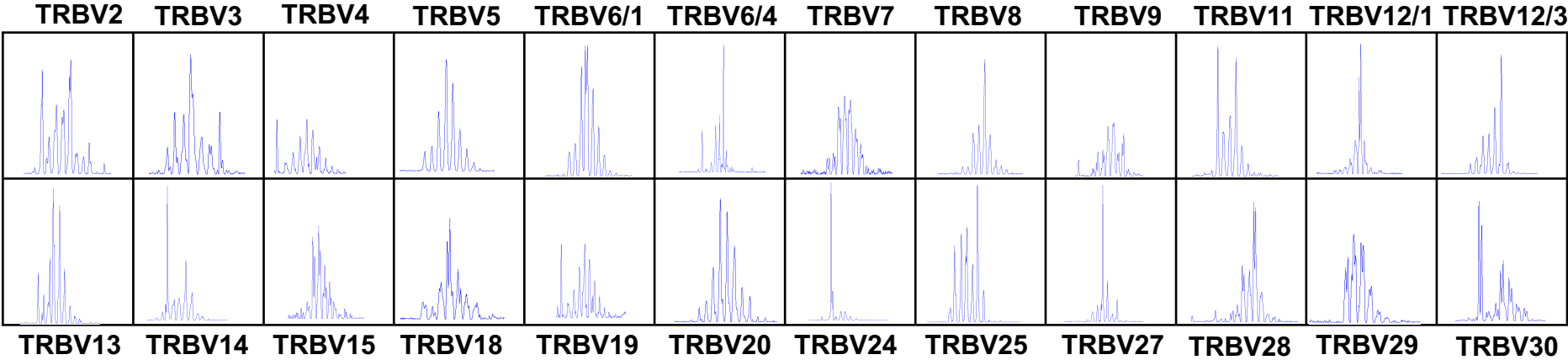

Supplement: Supplementary file 6 — Supplementary material 6 [file mmc6.pdf]

Figure E5

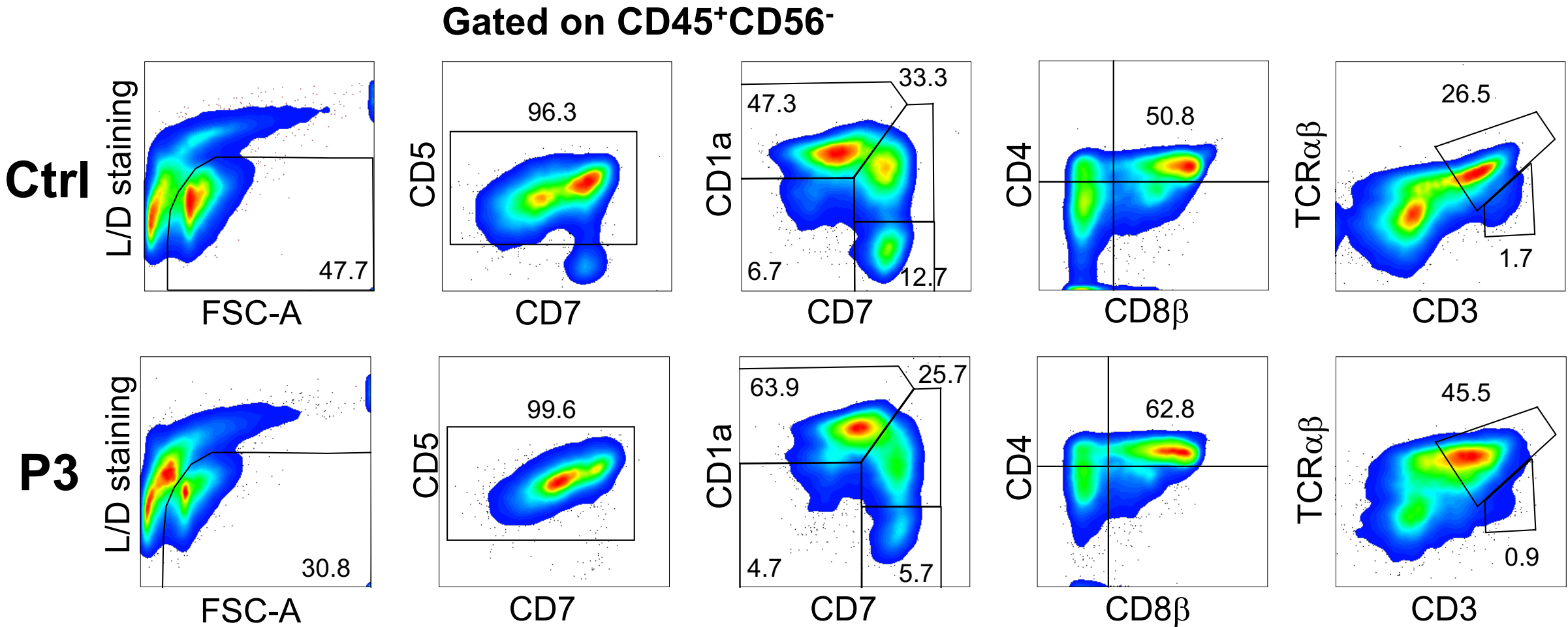

Supplement: Supplementary file 7 — Supplementary material 7 [file mmc7.pdf]
